# PSF-Driven Spatiotemporal Blending in Fluorescence Lifetime Imaging Microscopy and Its Mitigation via Mean-Shift Super-Resolution-Based Masking

**DOI:** 10.1021/acsphotonics.6c00741

**Published:** 2026-09-14

**Authors:** Mario González-Gutiérrez, Diana M. Vázquez-Enciso, Nicolás Mateos, Wonsang Hwang, Esley Torres-García, Haydee O. Hernández, Jenu V. Chacko, Iván Coto Hernández, Pablo Loza-Álvarez, Christopher Wood, Adan Guerrero

**Affiliations:** † Laboratorio Nacional de Microscopía Avanzada, Instituto de Biotecnología, Universidad Nacional Autónoma de México, Cuernavaca 62210, Mexico; ‡ Institut de Ciencies Fotoniques, Castelldefels 08860, Spain; § Wellman Center for Photomedicine, Harvard Medical School, Massachusetts General Hospital, CNY149, 13th Street, Charlestown Massachusetts 02129, United States; ∥ Facultad de Ingeniería, 56724 Universidad de la República, Montevideo 11300, Uruguay; ⊥ Centro de Investigación en Ciencias, 27783 Universidad Autónoma del Estado de Morelos, Cuernavaca 62209, Mexico; # Laboratory for Optical and Computational Instrumentation, University of Wisconsin at Madison, 1675 Observatory Dr., Madison Wisconsin 53706, United States; ¶ Center for Interdisciplinary Innovation in Imaging, 1811 Massachusetts General Hospital and Harvard Medical School, 149 13th Street, Charlestown Massachusetts 02129, United States; ∇ Centro Internacional de Ciencias AC. Cuernavaca 62210, Mexico

**Keywords:** fluorescence lifetime imaging microscopy, phasor analysis, mean-shift super-resolution, temporal blending, photon mixing, lifetime cross-talk

## Abstract

Fluorescence lifetime imaging microscopy (FLIM) enables quantitative mapping of molecular environments in living systems with high biochemical specificity. However, spatial overlap dictated by the diffraction-limited point spread function (PSF) causes a mixing of temporal signals: photons from neighboring emitters collected within the same pixel yield composite decay profiles, generating apparent intermediate lifetimes that can be mistaken for variations in the local molecular environment. We introduce a workflow that applies mean-shift super-resolution (MSSR) to raw intensity data to generate intensity-derived spatial masks prior to phasor-based lifetime analysis. The method is computationally efficient and preserves decay kinetics because it operates on intensity-derived spatial information rather than modifying temporal data. In U2OS cells labeled with spectrally-overlapping fluorophores, phasor analysis reveals an intermediate lifetime population localized at PSF-overlap interfaces, consistent with optical mixing rather than intrinsic lifetime heterogeneity. MSSR-derived masking suppressed this mixed population while preserving the distribution of similar phasor coordinates in the phasor plane for each fluorophore. Simulations of strictly monoexponential fluorescence decay emitters further show that blended lifetime decay profiles are present at separations up to 4σ, indicating that conventional spatial resolution criteria can underestimate lifetime cross-talk. Application of this workflow to three-component FLIM showed also a reduced overlap of pixel distributions in phasor plots while maintaining distinct lifetime signatures. Ultimately, the primary value of MSSR-based spatial refinement lies in leveraging improved spatial resolution to effectively isolate the temporal profiles of mixed fluorophore populations preserving the integrity of FLIM measurements.

## Graphical abstract

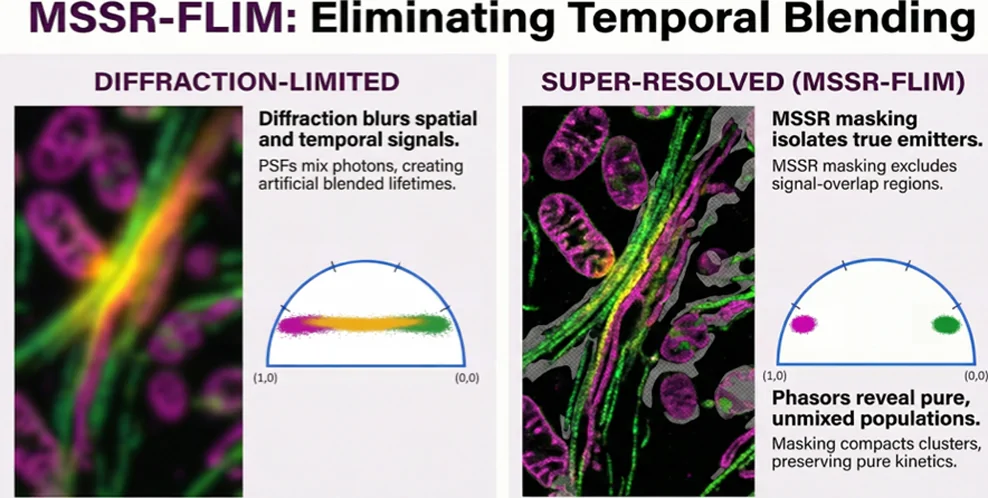

## Introduction

Fluorescence lifetime imaging microscopy (FLIM) maps the spatial distribution of fluorescence decay kinetics and thereby reports on biochemical and biophysical states that are often inaccessible to intensity-only imaging. By probing excited-state relaxation dynamics, FLIM enables contrast based on environmental sensitivity and molecular interactions even when fluorophore concentration, excitation inhomogeneity, or optical path variations confound intensity measurements. FLIM implementations are commonly classified into time-domain (TD-FLIM) and frequency-domain (FD-FLIM) modalities.^1–5^ TD-FLIM estimates lifetimes from the distribution of photon arrival times after pulsed excitation, most often using time-correlated single-photon counting (TCSPC) with single-photon detectors (including photomultiplier-based and single-photon avalanche diode (SPAD)-based detectors) or using widefield time-gated detection, and recent advances include analog approaches that record fluorescence waveforms.^1–5^ FD-FLIM, in contrast, derives lifetimes from the phase shift and demodulation of fluorescence emission signals relative to a temporally modulated excitation signal.^5^ FLIM has found extensive applications in medical imaging and in biochemical and physicochemical research, enabling quantitative assessments of parameters such as pH, viscosity, and other environmental or material properties.^3,6–16^

In FLIM measurements, uncertainty in the temporal characterization of fluorescence decays can arise from several distinct sources. First, the instrument response function (IRF) limits the temporal resolution with which photon arrival times can be resolved, imposing an instrumental broadening of the measured decay. Second, the stochastic nature of photon detection introduces shot-noise-limited uncertainty, particularly under low-count regimes.^17^ Third, and central to the present work, diffraction-limited imaging induces spatial convolution through the optical point spread function (PSF), such that photons emitted by neighboring fluorophores are collected within the same pixel. This spatial overlap produces composite decay signals that manifest as apparent lifetime mixtures that do not correspond to intrinsic photophysical interactions.^18,19^ We refer to this effect as PSF-induced temporal blending (TB).

Independent of acquisition modality, lifetime estimation can be performed using fitting or non-fitting approaches. Fitting methods include weighted least squares estimation (WLS), maximum likelihood estimation (MLE), and Global and Bayesian fitting. Non-fitting routines include rapid lifetime determination (RLD), Center-of-mass methods (CMM), Integral extraction methods, Laguerre Expansion, and the Phasor approach.^20^ More recently, machine learning and deep learning strategies have been explored to improve estimation accuracy and throughput, and to provide denoising capabilities.^21–24^

The phasor method provides a graphical representation of fluorescence decay kinetics by mapping each pixel to phasor coordinates (G,S). In this framework, monoexponential decays lie on the universal semicircle, which represents the geometric locus of phasor coordinates corresponding to lifetimes ranging from zero to infinity (for a given modulation frequency). Multiexponential decays fall inside this semicircle, where their position corresponds to the vector sum of the phasors of the individual lifetime components, weighted by their relative contributions. Intermediate phasor positions can reflect either genuine biochemical heterogeneity within the sampled volume or linear combinations of distinct lifetime components. This mixing property is useful for unmixing and segmentation, but it also implies that intermediate lifetimes can arise from optical cross-talk when photons from multiple emitters are pooled within a single spatial bin.^25–29^

As introduced above, PSF-induced temporal blending (TB) leads to spatially mixed decay signals that can bias lifetime estimation in diffraction-limited conditions. A direct strategy to mitigate TB is to increase spatial resolution so that neighboring emitters are better separated prior to lifetime extraction. Super-resolution techniques can be broadly classified into three categories. The first comprises deterministic approaches such as Stimulated Emission Depletion (STED) and Structured Illumination Microscopy (SIM).^30–35^ STED has been integrated with lifetime measurements through multiple strategies, including time-gated STED, Separation of Photons by Lifetime Tuning (SPLIT-STED), STED-FLIM combined with phasor analysis, and TauSTED, which exploit depletion-induced lifetime gradients to refine contrast and spatial information.^36–43^ The second category comprises stochastic localization-based super-resolution methods collectively referred to as Single-Molecule Localization Microscopy (SMLM). Representative SMLM implementations include Photoactivated Localization Microscopy (PALM) and Stochastic Optical Reconstruction Microscopy (STORM), which reconstruct super-resolved images by localizing temporally separated single-emitter signals over many acquisition frames.^44–46^ Lifetime-resolved variants, including confocal fluorescence-lifetime SMLM and time-correlated SMLM, further increase molecular specificity by assigning lifetime values to localized molecules using TCSPC-based detection.^47,48^

The third category comprises computational approaches, including deconvolution methods that attempt to reverse optical blurring using an estimated PSF, as well as fluctuation-based super-resolution methods such as cross-cumulant Superresolution Optical Fluctuation Imaging (XC-SOFI), Bayesian analysis of Bleaching and Blinking data (3B), Entropy-based Superresolution Imaging (ESI), and Super-Resolution Radial Fluctuations (SRRF).^46^ Addressing TB specifically, Bastiaens and Squire developed an iterative constrained deconvolution method based on the Tikhonov–Miller algorithm (ICTM), processing temporal information through Fourier components that are trigonometric analogs of the phasor coordinates (G,S).^18,49^ While effective in relevant contexts, many computational super-resolution strategies require large frame stacks, substantial computation, or temporal processing steps that may introduce artifacts or confounds when applied to FLIM, particularly when preservation of decay kinetics is essential.^17^

Mean-shift super-resolution (MSSR) offers a distinct opportunity for FLIM because it operates on intensity information to enhance spatial separability without directly altering the fluorescence decay kinetics.^50^ In the present workflow, MSSR is applied to an average (or integrated) intensity projection of the FLIM data set to generate an intensity-derived spatial mask with improved separability and signal-to-noise ratio (SNR). Phasor coordinates are then computed from the unmodified time-resolved FLIM data within the masked regions, thereby suppressing PSF-driven photon mixing while conserving the lifetime signatures. Accordingly, we developed and validated a method that couples MSSR-derived spatial masks with phasor analysis, demonstrating that improved spatial separability can reduce temporal blending in FLIM while maintaining decay fidelity in complex specimens.

## Results

The central hypothesis of this work is that intermediate apparent lifetime signatures can arise from optical mixing rather than intrinsic fluorescence heterogeneity. A brief conceptual foundation in phasor analysis is therefore provided to support interpretation of the results (Supporting Information, Figure S1). In phasor space, monoexponential fluorescence decays map onto the universal semicircle, whereas multicomponent decays and mixtures of fluorescence signals occupy positions within the semicircle according to the linear-combination rule. Consequently, when photons emitted by distinct monoexponential species are collected within the same diffraction-limited observation volume, the resulting phasor lies at an intermediate position between the corresponding end point lifetimes.

### Spatial Convolution Drives Temporal Blending in Lifetime Imaging

To experimentally illustrate how temporal blending (TB) emerges at the cellular scale, we analyzed a fixed-cell specimen in which two distinct subcellular structures—mitochondria and microtubules—were labeled with spectrally overlapping fluorophores. Alexa Fluor 532 (AF532; TOM20) and Alexa Fluor 555 (AF555; α-tubulin) have closely spaced emission maxima (553 and 580 nm), rendering the two structures indistinguishable in single-channel confocal intensity imaging (Figure 1a). In this reference specimen, both dyes are immobilized via immunolabeling on their respective targets, and no direct biochemical coupling between labels is expected. The system therefore approximates two independent lifetime species; under this condition, intermediate apparent lifetimes are predicted to arise predominantly from optical photon pooling when diffraction-limited point spread functions (PSFs) overlap.

**Figure 1:**
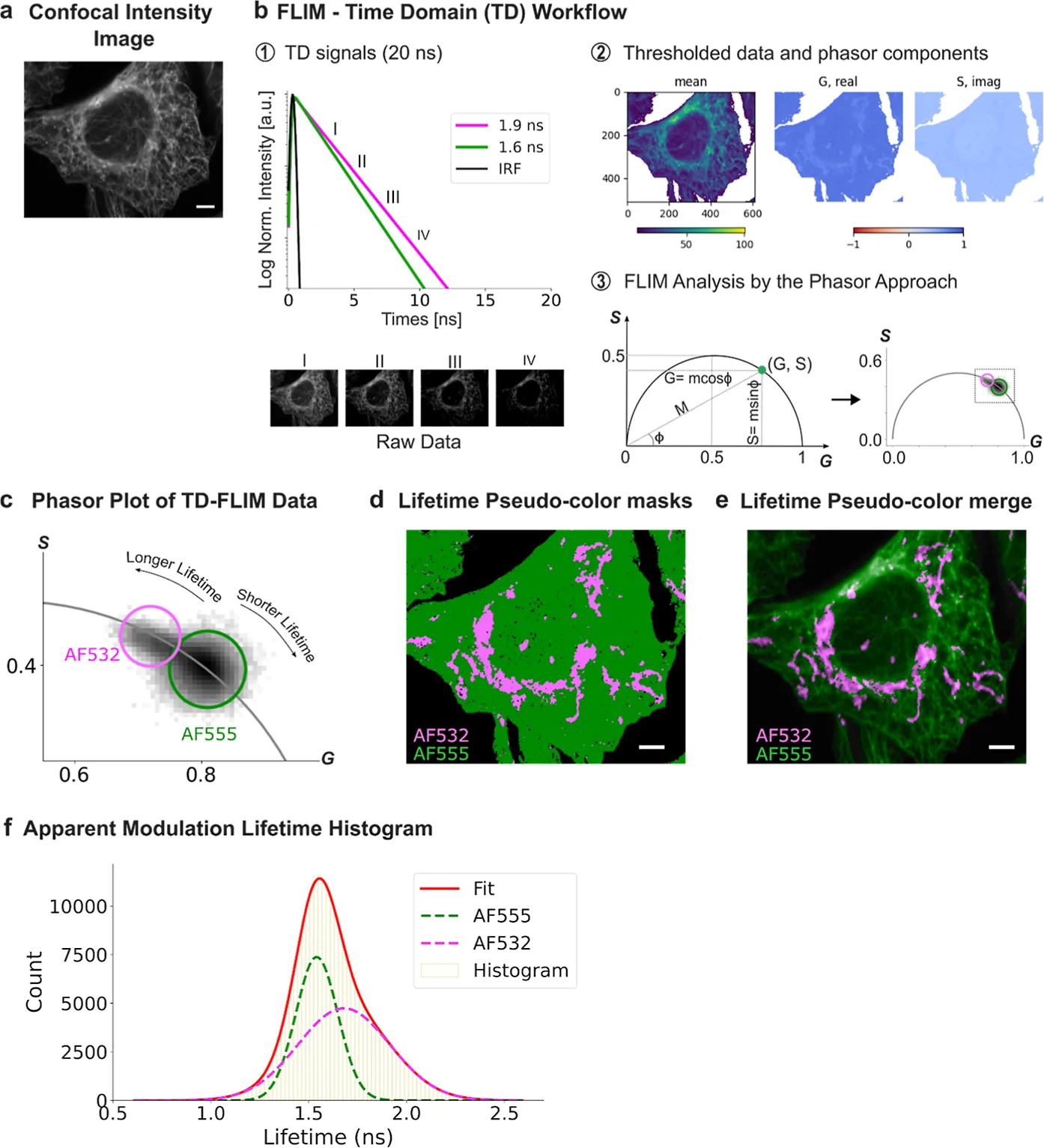
TD-FLIM phasor workflow and end point lifetime populations in a dual-labelled cellular specimen. (a) Single-channel confocal intensity image of a U2OS cell specimen labeled for mitochondria (AF532; targeting protein TOM20) and microtubules (AF555; targeting α-tubulin peptides); structures are not distinguishable in the intensity domain due to spectral proximity. (b) TD-FLIM workflow schematic. (b1) Each pixel contains a time-resolved intensity profile; schematic monoexponential decays illustrate two independent pixels dominated by different fluorophore species (short- and long-lifetime exemplars), corresponding to the qualitative end point behaviors expected for AF555- and AF532-enriched regions in this specimen. Traces are schematic and included for conceptual reference rather than dye-specific calibration. (b2) Mean intensity image and phasor-component images (G and S) after intensity thresholding. (b3) Phasor representation schematic: a single-component decay maps to a point on the universal semicircle (left), whereas the experimental data exhibit two lobes (right), displayed in detail in (c). (c) Phasor plot of experimental TD-FLIM data with two end point cursor selections defining AF532-enriched and AF555-enriched populations. (d) Pseudo-color lifetime mask obtained by projecting cursor selections from (c) into image space. (e) Pseudo-color mask merged with intensity. (f) Apparent modulation lifetime histogram for end point-selected populations with Gaussian fits. Scale bar: 2.7 μm.

Figure 1 summarizes the TD-FLIM phasor workflow used to evaluate TB in the cellular specimen. Each pixel contains a temporal-domain intensity profile sampled across time gates, and the full set of pixel-wise decays is analyzed to compute phasor-component images (G and S) and a mean intensity image after intensity thresholding (Figure 1b2). Figure 1b1 shows schematic monoexponential decay exemplars for two independent pixels dominated by different fluorophore species, illustrating the end point behaviors expected for a two-species specimen (schematic traces are included for conceptual reference rather than dye-specific calibration under the present conditions). Phasor coordinates derived from all pixels populate the phasor plot (Figure 1b3,c), and the reciprocity between phasor space and image space enables bidirectional mapping: cursor selections in phasor space can be projected back into the image to generate spatial masks, and spatial regions of interest can be mapped to their corresponding phasor distributions.^27^

The experimental phasor distribution exhibited two dominant lifetime populations (Figure 1c). Rather than forming two fully separated compact clusters, the data displayed two partially separated lobes connected by an elongated continuum between the end point regions, consistent with pixels containing composite decays. Two circular cursors were placed at the ends of the distribution to define end point populations enriched in the short- and long-lifetime components. Projecting these end point selections back into image space generated pseudo-color lifetime masks (Figure 1d) that segmented microtubules predominantly into the short-lifetime class (green; AF555-enriched) and mitochondria predominantly into the long-lifetime class (pink; AF532-enriched). The merged pseudo-color/intensity representation (Figure 1e) preserved this spatial organization across the cell while highlighting interface zones where class separation became locally heterogeneous, consistent with spatial mixing.

To quantify the end point populations, apparent modulation lifetime histograms were computed from the pixels selected by the end point cursors (Figure 1f). The histogram was described by two main components corresponding to the end point classes. The short-lifetime end point population (AF555-enriched; green) appeared comparatively narrow, whereas the long-lifetime end point population (AF532-enriched; pink) was broader, indicating increased dispersion in apparent lifetimes within the long-lifetime class. This broadening suggested that a fraction of pixels assigned to the end point classes still contained mixed contributions, motivating explicit identification of intermediate phasor positions expected under the optical mixing behavior introduced in Figure S1.

To localize intermediate apparent lifetimes, a third cursor was placed on the central region between the end point lobes (Figure 2a). This selected isolated pixels whose phasor coordinates fell between the end point populations, consistent with composite decays. Mapping these intermediate pixels back into image space revealed preferential localization at interfaces between mitochondria and microtubules (Figure 2b), where PSF overlap and photon pooling from adjacent emitters are expected to be maximal. The merged pseudo-color representation (Figure 2c) further emphasized that the intermediate class (yellow; TB) was spatially confined and enriched at boundary and crossing regions rather than uniformly distributed within either structure. Representative regions of interest (ROI 1–3) highlight the characteristic spatial motif of TB pixels decorating mitochondria–microtubule appositions.

**Figure 2:**
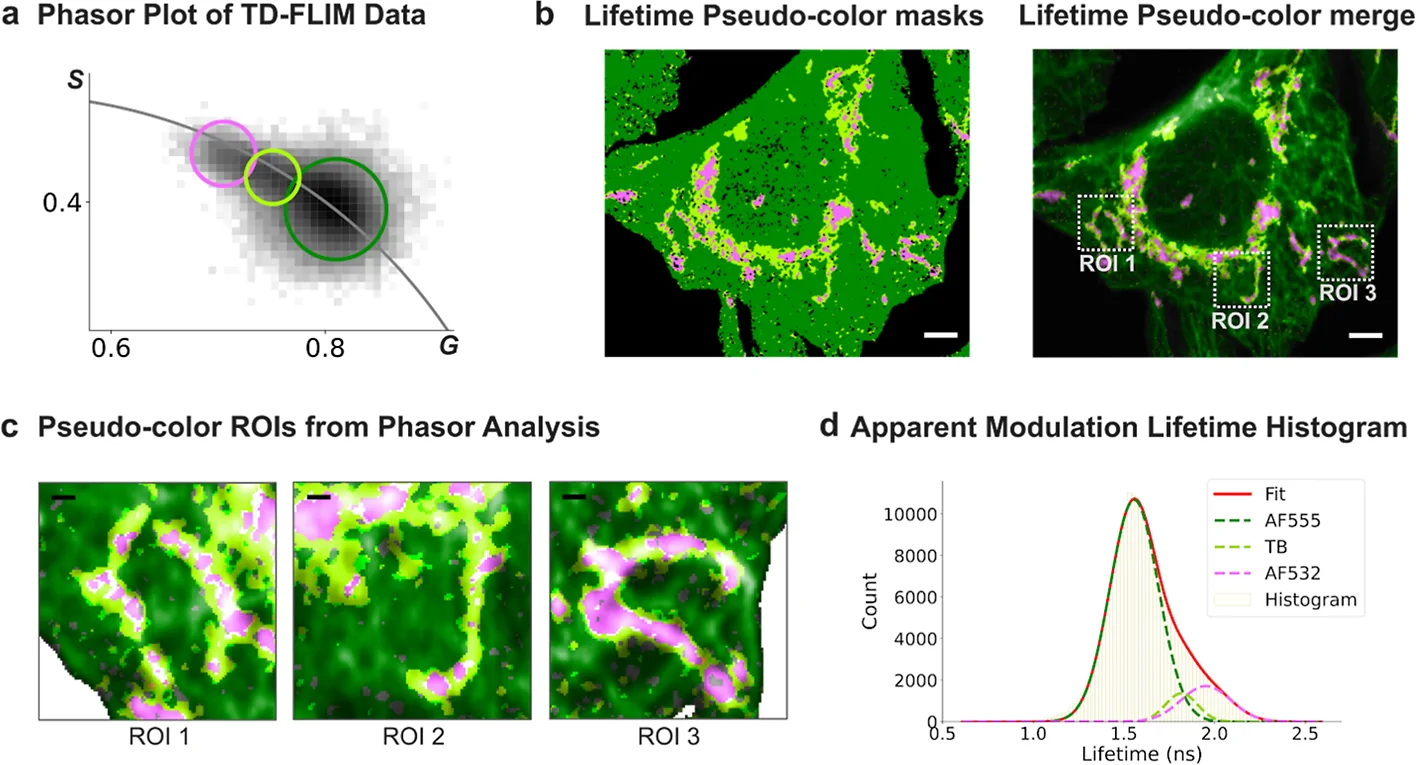
Temporal blending manifests as an intermediate phasor population localized at mitochondria–microtubule interfaces. (a) Phasor plot showing end point populations (AF532-enriched, pink; AF555-enriched, green) and an intermediate population (yellow) selected by a third cursor positioned between end point lobes. (b) Pseudo-color segmentation map obtained by projecting the three cursor selections into image space, highlighting the intermediate population at structural interfaces. (c) Pseudo-color merge with intensity overlay and three representative regions of interest (ROI 1–3) illustrating interface-localized intermediate pixels. (d) Apparent modulation lifetime histogram computed from the three pixel classes with a triple-Gaussian fit separating AF555-enriched, AF532-enriched, and TB-associated intermediate components. Scale bars in (b) 3 μm; scale bars in (c) 500 nm.

Quantitatively, adding the intermediate pixel class revealed an apparent modulation lifetime distribution that was best described by three components (Figure 2d): a short-lifetime end point (AF555-enriched), a long-lifetime end point (AF532-enriched), and an intermediate component assigned to TB. Because both fluorophores are immobilized by immunolabeling on distinct, non-coupled targets (α-tubulin versus TOM20), a third interaction-derived lifetime population (for example, FRET or chemically-coupled photophysics) is not expected at the scale of these structures. Instead, an intermediate apparent lifetime is predicted where diffraction-limited sampling volumes pool photons from both labels, yielding an amplitude-weighted composite decay that falls between end points in the phasor space. The phasor continuum linking end point lobes (Figure 1c), the interface-enriched localization of intermediate pixels (Figure 2b,c), and the intermediate peak resolved by three-component fitting (Figure 2d) together support PSF-driven photon mixing as the source of spatially patterned intermediate lifetimes, establishing a baseline for evaluating masking strategies that improve spatial separability while preserving end point signatures.

## Mean-Shift Super-Resolution Mitigates Temporal Blending in Fluorescence Lifetime Imaging Microscopy

The baseline temporal blending (TB) signature established above, defined as a spatially patterned intermediate lifetime class concentrated at structural interfaces, led us to explore a strategy that increases spatial separability without direct manipulation of decay kinetics. Mean-shift super-resolution (MSSR) operates on the spatial intensity landscape and can reduce effective PSF overlap through redistribution of signal density toward local maxima, thereby improving emitter separability in diffraction-limited data.^46,50,51^ In the present workflow, MSSR is used as a spatial prior that defines where photons are likely to originate, after which lifetime information is interpreted from the original time-resolved measurements. This ordering is essential: spatial processing is applied to an intensity projection to derive a mask, and phasor coordinates remain derived from the unmodified time domain FLIM data, preserving the decay information that underpins the phasor representation.

A practical aspect of this implementation is that the masking step uses an intermediate-bounded output from MSSR rather than the final remapped intensity image. Specifically, the procedure intentionally omits the last MSSR intensity remapping (normalization step) and instead uses the intermediate matrix that is naturally bounded between 0 and 1, denoted here as ( $P_{MSSR^{n}}$ ). This quantity behaves as a probability-like spatial support map, i.e., values near 1 concentrate around likely emitter locations, whereas low values occupy background and overlap regions. Thresholding $P_{MSSR^{n}}$ therefore yields binary masks that progressively restrict analysis to spatial regions with high localization likelihood, a property exploited here to mitigate mixed-pixel signatures while maintaining the end point lifetime classes identified previously.

The expected effect of MSSR on separability is illustrated using a two-emitter model at the Sparrow limit (two Gaussian emitters separated by 2σ). In the raw summed profile, strong overlap yields a single broadened maximum and poor resolvability at the midpoint (Figure 3a). After MSSR processing, the probability-like output becomes increasingly bimodal as MSSR order increases, enabling threshold selection that isolates high-support cores of each emitter while suppressing the overlap region (Figure 3b). The corresponding two-dimensional maps illustrate that, across threshold levels, raw data retain substantial overlap, whereas MSSR^0^ and MSSR^1^ progressively concentrate probability density into separated lobes (Figure 3c). These model results define the operational principle used in the cellular experiment. Figure 4 applies the same $P_{MSSR^{n}}$ -thresholding logic outlined in Figure 3 to generate a spatial mask from a FLIM intensity projection, with the goal of suppressing overlap-dominated pixels that are predicted to underline intermediate phasor positions.

**Figure 3:**
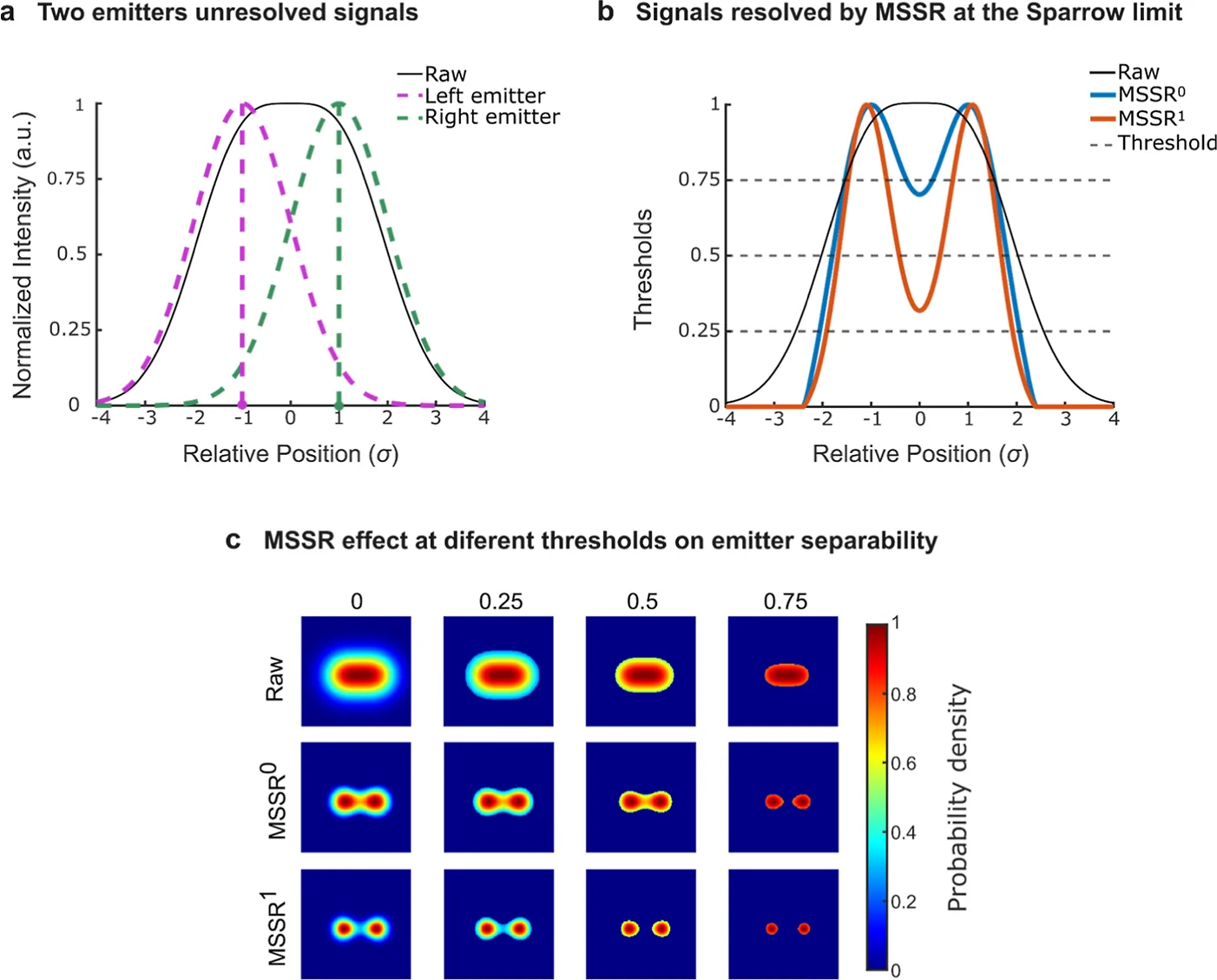
Resolution enhancement by MSSR at the Sparrow limit using a probability-like intermediate output (*P*_MSSR_). (a) Two Gaussian emitters separated by 2σ (Sparrow criterion) shown as individual contributions (magenta dashed, left emitter; green dashed, right emitter) and their summed signal (black). The summed profile is effectively unresolved at the midpoint under this separation. (b) One-dimensional profiles after MSSR processing, displayed as the intermediate bounded output used in this work P_MSSR_; computed prior to the final MSSR intensity remapping step. Raw (black), MSSR^0^ (blue), and MSSR^1^ (red) are shown. Horizontal dashed lines indicate representative threshold levels applied to *P*_MSSR_ to restrict support to high-probability regions. (c) Two-dimensional probability-density maps illustrating the combined effect of MSSR order (rows: Raw, MSSR^0^, MSSR^1^, and threshold level (columns) on emitter separability. Color bar indicates probability density (0–1). The horizontal axis in (a,b) is expressed in units of σ, the standard deviation of the Gaussian distributions used in panel (a).

**Figure 4:**
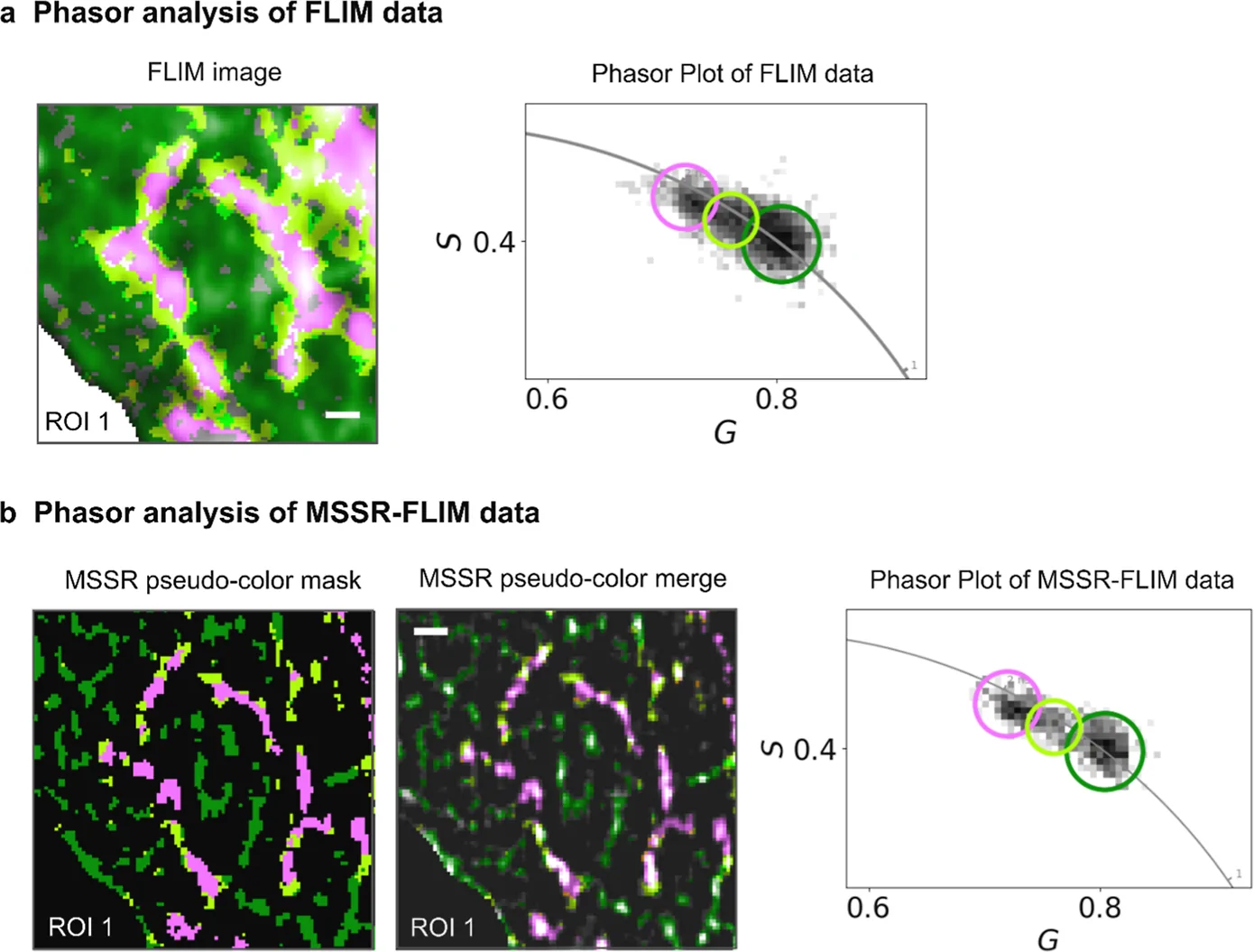
MSSR-derived probability masking reduces mixed-pixel signatures in phasor space without shifting end point lifetime clusters. (a) Conventional FLIM analysis of ROI 1. Phasor-based pseudo-color map and the corresponding phasor plot (right), showing two end point-enriched populations connected by an intermediate population. Circular cursors define the pseudo-color classes (green and magenta for end point-enriched populations; yellow for the intermediate/mixed pixels). (b) MSSR-FLIM analysis. A MSSR-derived probability-like map $P_{MSSR^{1}}$ generated from the FLIM intensity projection is thresholded to form a binary mask that is applied to the phasor-coordinate images (G,S) computed from the original time-resolved FLIM data (left). The retained phasor information is merged with the MSSR-enhanced intensity image to generate the pseudo-color merge (center). The resulting phasor distribution preserves the end point populations while reducing the intermediate mixed-pixel population, leading to improved spatial separation of the lifetime signatures in ROI 1. Quantitative metrics describing centroid displacement and phasor cluster compactness are reported in the main text. Scale bar: 500 nm.

To test this prediction in the cellular specimen, MSSR was applied to an intensity projection of the FLIM acquisition to generate $P_{MSSR^{1}}$ , which was thresholded to produce a binary mask. The mask was then applied to the phasor-coordinate images (G,S) computed from the original time-resolved FLIM data, so that only spatially supported pixels contributed to the phasor plot and to the corresponding pseudo-color maps (Figure 4b). If MSSR preprocessing had distorted decay kinetics, such distortion would manifest as a systematic displacement of the end point phasor clusters. Instead, the masked analysis preserves the end point *loci* while altering the spatial sampling of pixels that enter the analysis (compare Figure 4a versus Figure 4b), consistent with conservation of the underlying lifetime signatures. This invariance was quantified by computing phasor-cluster centroids before and after masking and measuring their displacement as a Euclidean distance in (G,S) space; centroid shifts were minimal (0.0066 for the whole cell and 0.0064 for ROI 1) and remained well below a conservative no-distortion bound of 0.05 phasor units selected to exceed fluctuations expected from photon noise and finite sampling (Figure 4a,b; Methods). In parallel, the spatial maps show improved delineation of microtubule-like and mitochondria-like structures within the ROI, accompanied by an apparent reduction in the number of pixels assigned to the intermediate mixed class (Figure 4b, ROI 1). Consistent with reduced mixing, the phasor clusters obtained after masking exhibit increased compactness, which was quantified using an ellipse-area ratio defined as the cluster ellipse area after masking divided by the area before masking, which remained below unity for end point clusters (Methods). Because reduced phasor cluster area reflects diminished pooling of heterogeneous decays within single pixels, the combined behavior of minimal centroid displacement and reduced ellipse area supports the conclusion that MSSR-derived probability masking mitigates TB-associated mixing signatures while preserving the end point lifetime components of the specimen. In other words, it reduces intermediate apparent lifetimes attributable to photon mixing.

### In Silico Characterization of Diffraction-Induced Temporal Blending Evidenced as Phasor Mixing

To systematically characterize TB, we developed an in silico model composed of two independent, noninteracting fluorophores. This simulation isolates and quantifies the contribution of point spread function (PSF) overlap to TB at controlled spatial separations (Figure 5). The model is applicable to both time-domain FLIM (TD-FLIM, Figure 5a) and frequency-domain FLIM (FD-FLIM, Figure S3) modalities.

**Figure 5:**
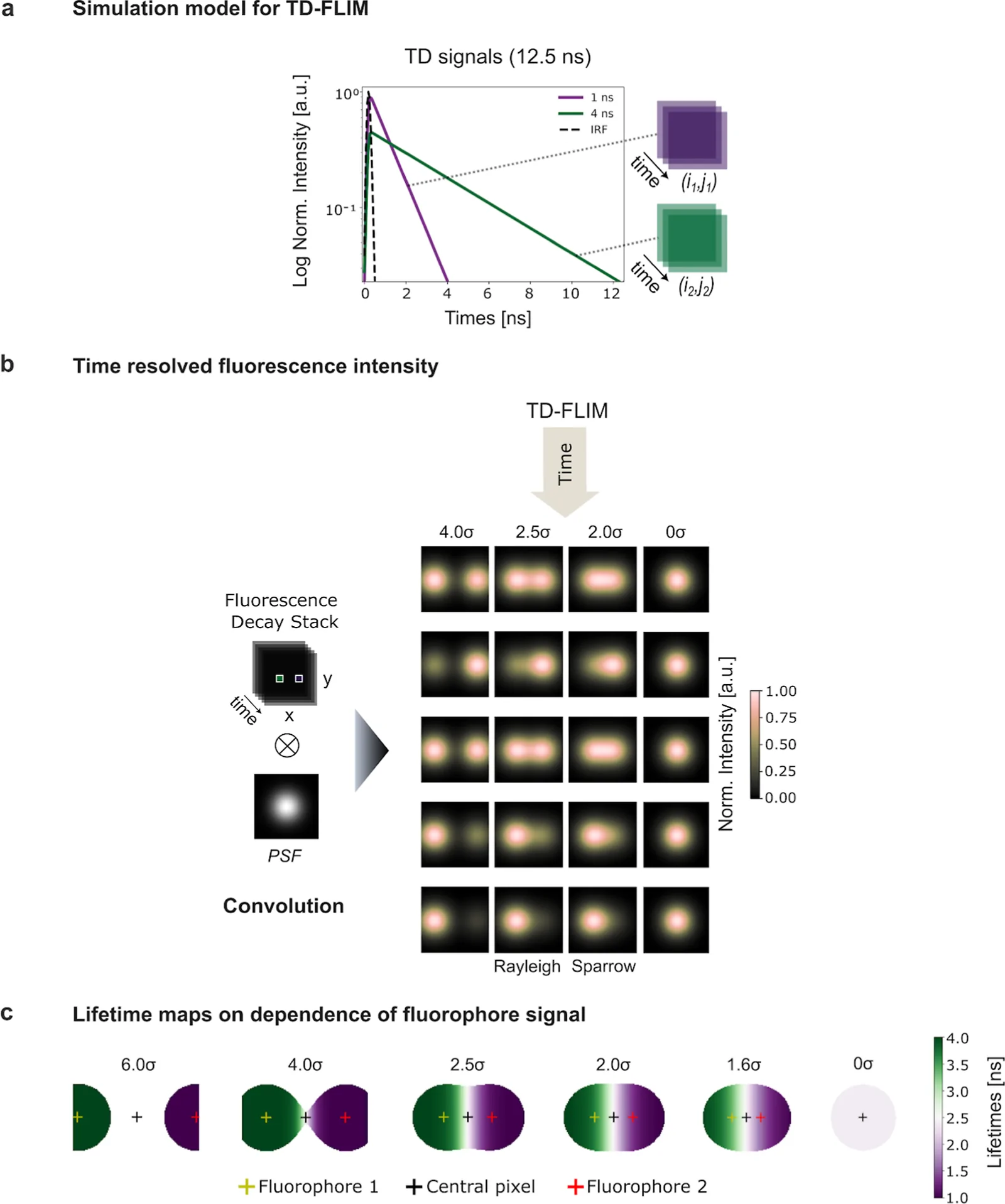
Simulation framework for characterizing temporal blending in FLIM. (a) Schematic representation of the in silico model for two independent, non-interacting fluorophores in TD-FLIM. The simulated time-varying fluorescence signal of each fluorophore is stored in a single pixel at a defined position (*i*_*k*_,*j*_*k*_). (b) To account for diffraction-limited spatial blurring, both pixels are convolved with a Gaussian point spread function (PSF), yielding time-resolved image stacks; representative frames are shown for separations of 4.0σ, 2.5σ, 2.0σ, and 0σ. (c) After temporal analysis using the Fourier-based phasor workflow, pseudo-color lifetime maps are generated for separations from 6σ to 0σ, showing progressive emergence of intermediate apparent modulation lifetimes in the overlap region. Crosses indicate fluorophore positions (fluorophore 1, long-lived; fluorophore 2, short-lived) and the central reference pixel (empty unless the separation is 0σ).

Building on an initial frequency domain approximation of signal mixing,^52^ we implemented a more general simulation framework that explicitly accounts for PSF-induced mixing prior to phasor extraction. This refinement avoids a coarse-grained representation of temporal behavior and enables the same simulation backbone to be used in both the TD-FLIM and FD-FLIM regimes. Using PhasorPy,^53^ time-resolved fluorescence signals were generated for two fluorophores with distinct lifetimes of 1 ns and 4 ns. The temporal information was initially assigned to a single pixel for each fluorophore, namely, fluorophore 1 (long-lived, 4 ns) at pixel (*i*_1_,*j*_1_) and fluorophore 2 (short-lived, 1 ns) at pixel (*i*_2_,*j*_2_) (Figure 5a). A time-resolved image stack with dimensions (*x*, *y*, *t*) was then constructed, where each frame *t* represents a discrete time point within the simulated excitation cycle. To model spatial blurring, the fluorophore pixels were convolved with a two-dimensional Gaussian PSF (Figure 5b).

Figure 5b illustrates the time-varying fluorescence intensities in TD-FLIM regime and the corresponding blurred frames for representative separations (4.0σ, 2.5σ, 2.0σ, and 0σ). In this regime, intensity decreases monotonically across frames. After temporal analysis of each simulated condition using the same Fourier-based phasor workflow described in the Methods and illustrated in Figure 6, pseudo-color lifetime maps were generated for six spatial separations ranging from 6σ to 0σ (Figure 5c). When the fluorophores are well separated, pixels within each PSF report the ground-truth lifetimes (1 ns and 4 ns). As the fluorophores approach one another, PSF overlap increases and lifetime values blend in the overlapping region, producing intermediate apparent lifetimes. In the case of complete spatial overlap (0σ), the phasor representation collapses toward a single amplitude-weighted mixed point, yielding a spatially uniform apparent lifetime in the derived map.

**Figure 6:**
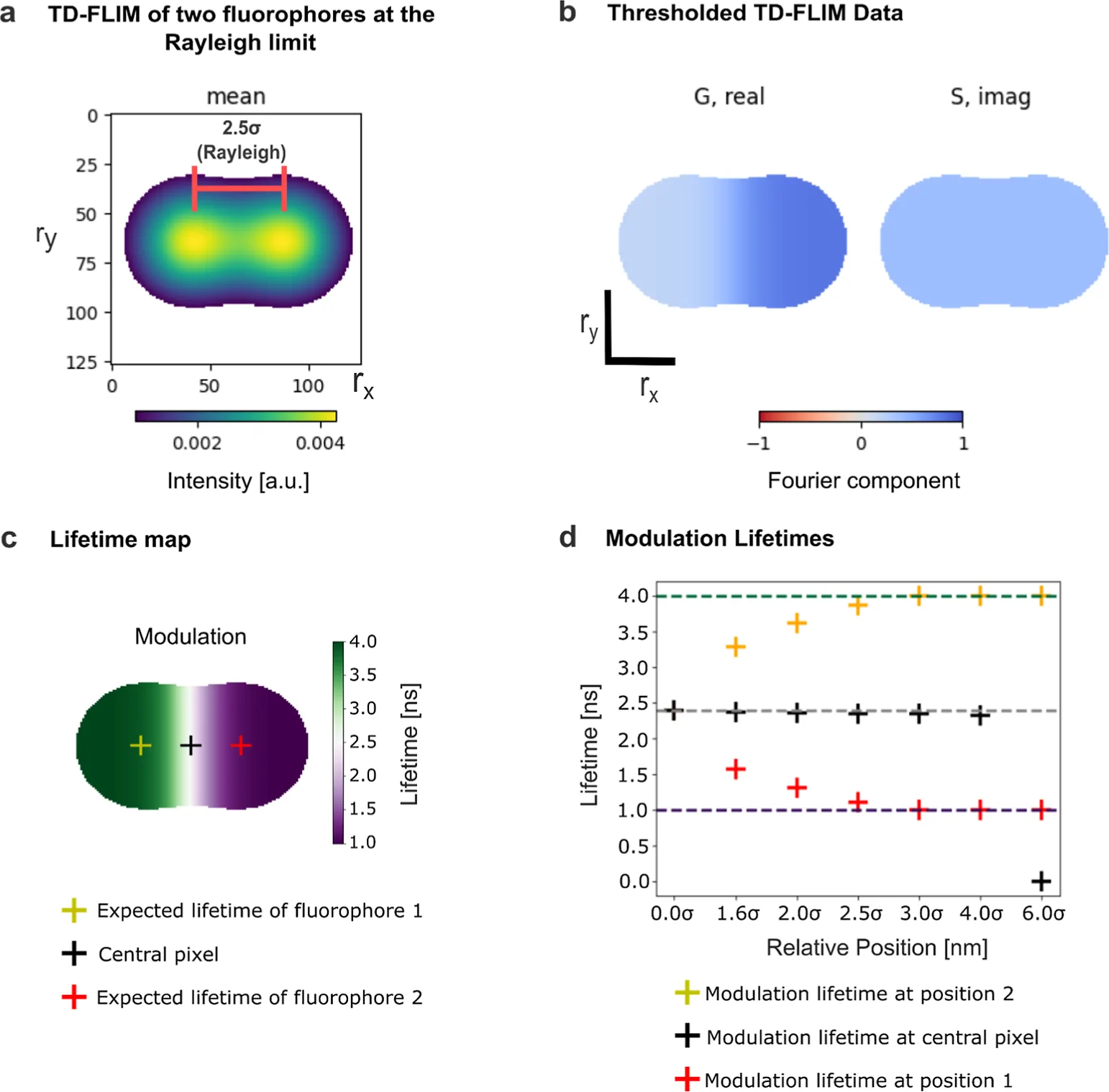
Temporal blending in FLIM arises from PSF-induced spatial blurring. Panels (a–c) show simulated TD-FLIM images of two fluorophores positioned at 2.5σ. (a) Steady-state fluorescence intensity (mean from Fourier analysis) with intensity thresholding applied to suppress background. Axes represent pixel coordinates (*r*_*x*_,*r*_*y*_). (b) Phasor component maps: G (real) and S (imaginary). (c) Apparent modulation lifetime map computed from Fourier components. (d) Modulation lifetimes sampled at the centers of each fluorophore position and at the central reference pixel, plotted as a function of inter-fluorophore separation (σ).

For the lifetime analysis, each simulated FLIM stack was processed using Fourier decomposition to transform temporal fluorescence signals into phasor space (Figure 6). The time-invariant component corresponds to the steady-state fluorescence (mean intensity), illustrated for the case of two fluorophores positioned at 2.5σ (Figure 6a). Phasor coordinates were computed using the Fourier-based implementation in PhasorPy.^53^ Background contributions were suppressed by applying an intensity threshold to the mean intensity map (Figure 6a).

Phasor component maps (Figure 6b) revealed a smooth gradient in the G component between the two simulated fluorophores rather than a sharp boundary, consistent with PSF-driven mixing of temporally distinct signals. The corresponding modulation lifetime map (Figure 6c) further confirms this effect through the emergence of intermediate apparent lifetimes in the overlap region. Notably, the phase lifetime map displays a shift in the transition zone toward the long-lifetime fluorophore (Supporting Information Figure S2), suggesting a similar effect to the Frequency Modulation Capture Effect (FMCE) described by Yuan, X. et al.^54^ Finally, modulation lifetime values sampled at the centers of the fluorophore positions and at the central reference pixel were quantified as a function of separation (in units of σ) (Figure 6d). A clear dependence on inter-fluorophore separation was observed, highlighting the role of PSF overlap in driving TB in FLIM measurements. The same framework was used to study TB in the FD-FLIM simulations (Supporting Information Figure S3).

TB is present in pseudo-color lifetime maps even at an inter-fluorophore distance of 4σ (Figure 7). The case at 1.6σ is particularly informative because this separation has been reported as a practical MSSR resolution limit.^50^ As the separation decreases from well-resolved to fully overlapped, the phasor cluster broadens and shifts toward intermediate G values, consistent with an amplitude-weighted phasor mixture produced by PSF overlap and photon pooling, which yields apparent intermediate lifetimes in the derived maps. In the fully overlapped case (0σ), the phasor distribution collapses to a single point corresponding to the mixture of both components. At the opposite extreme (6σ), only one pixel is sampled for each lifetime as the modeled signal arises solely from the two discrete fluorophores with no additional contributions.

**Figure 7:**
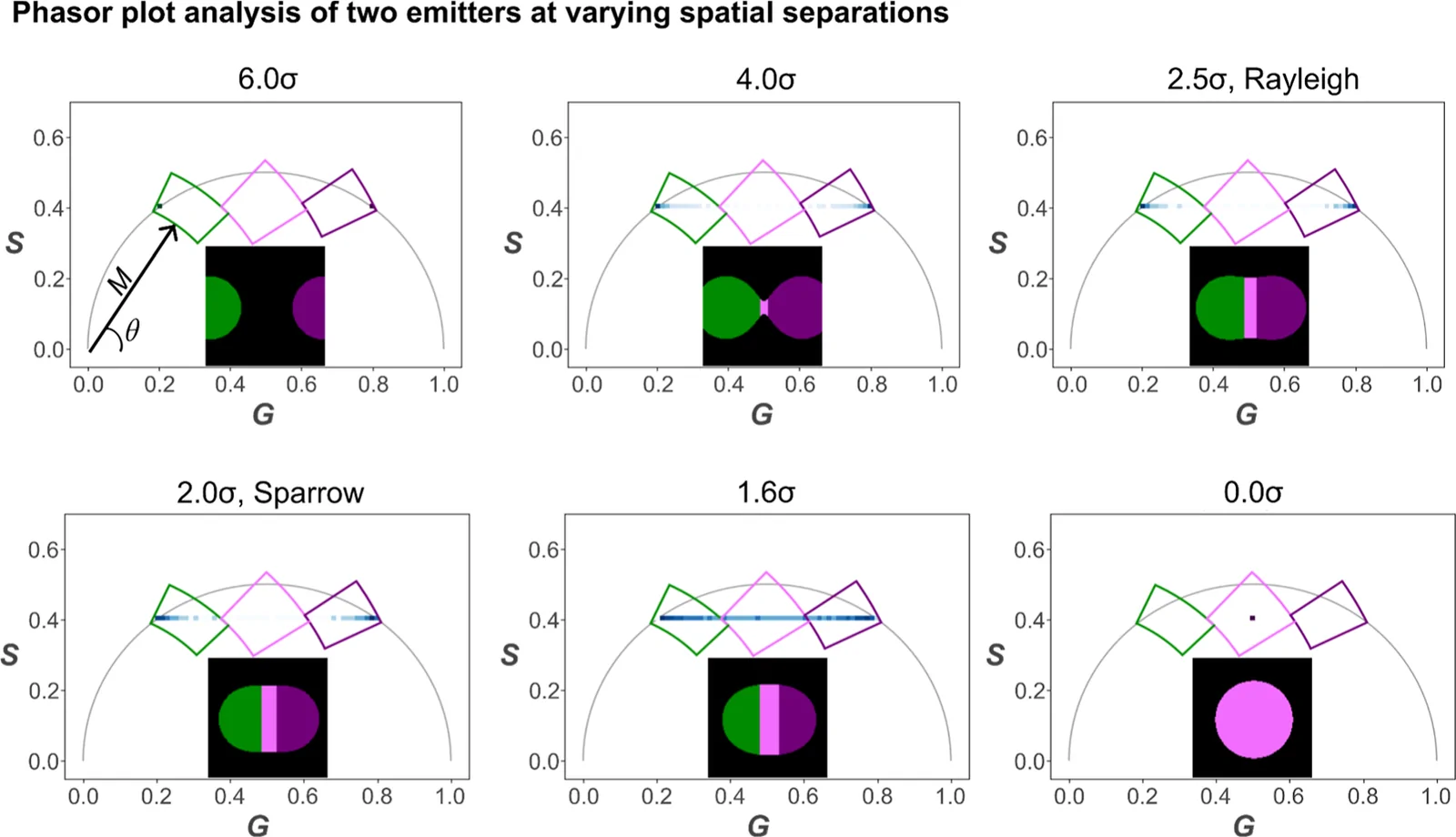
Phasor analysis across inter-fluorophore separations reveals progressive broadening and migration of mixed-pixel signatures. Each panel shows the phasor distribution (G, S) for a given separation (in units of σ) with the universal semicircle. Rectangle-polar cursor selections illustrate how phasor-space regions map, via reciprocity, to spatially distinct regions in the corresponding pseudo-color lifetime maps (insets). Increased PSF overlap produces intermediate phasor positions consistent with temporally mixed pixels.

Following the approach described above, MSSR was applied to reduce TB and enhance spatial resolution for the challenging case of two emitters positioned at the Sparrow diffraction limit (2σ), as shown in Figure 8. As illustrated in Figure 8a, the raw intensity image exhibits substantial PSF-induced signal overlap, which manifests in phasor space as an extended distribution characteristic of mixed pixels (Figure 8a, right). Cursor selection was used to isolate pixels corresponding to monoexponential decays, operationally defined as phasor components lying along the universal semicircle.

**Figure 8:**
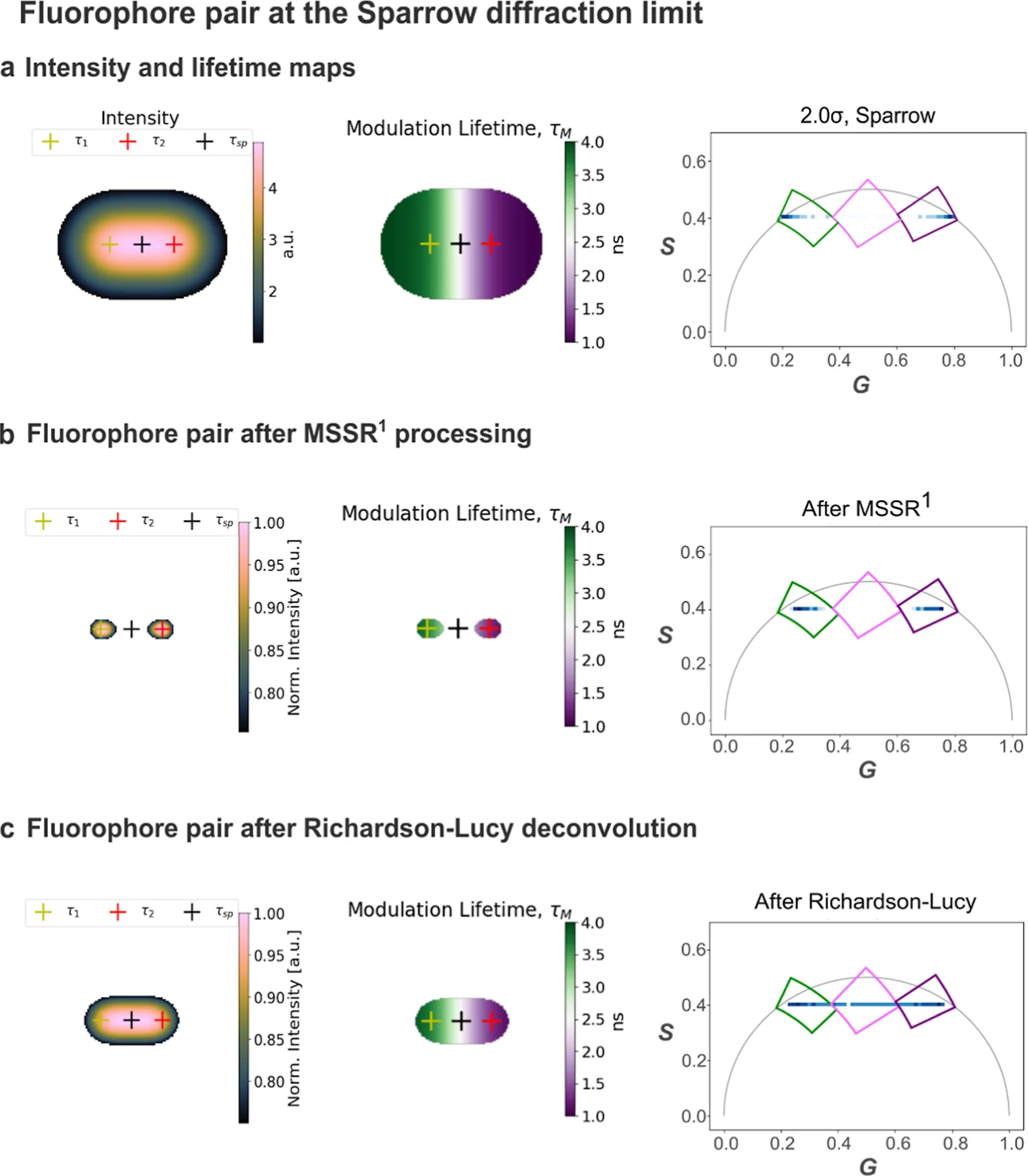
Mitigating temporal blending using mean-shift super-resolution: a computational approach. Two independent, non-interacting simulated fluorophores are positioned at the Sparrow limit (2.0σ; Figure 3). (a) Raw condition: intensity profile (left), modulation lifetime map (middle), and phasor plot (right) showing an extended distribution characteristic of mixed pixels; cursor selection highlights monoexponential components along the universal semicircle. (b) MSSR masking applied to the data set in (a) prior to phasor transformation using the 75th-percentile threshold (see Figure S4 for threshold comparison). The MSSR-masked analysis increases spatial separation and compacts the phasor distribution, indicating reduced temporal blending while preserving the component decay signatures; compaction is quantified by the ellipse area ratio and centroid displacement in (G, S) units. (c) Richardson-Lucy deconvolution followed by masking of the data set shown in (a) using the 75th-percentile threshold prior to phasor transformation. Compared with the MSSR-based workflow, the modulation lifetime image exhibits less complete separation of the fluorophore signals. Accordingly, the corresponding phasor plot retains a continuous bridge of intermediate phasor populations between the end point clusters, consistent with residual PSF-mediated optical mixing.

The associated modulation lifetime map (Figure 8a, middle) reveals a spatially continuous intermediate lifetime region between the two emitters, reflecting temporal blending arising from spatial averaging. MSSR was then applied as a spatial probability mask derived from the fluorescence intensity distribution, using percentile-based thresholds (25th, 50th, and 75th) to progressively isolate regions with a high likelihood of originating from individual emitters; each threshold mask was applied prior to the phasor transformation (Supporting Information Figure S4).

Figure 8b shows the result obtained using the 75th-percentile threshold. Under this Sparrow-limit condition, the MSSR markedly improves spatial separability, as evidenced in the modulation lifetime map by the collapse of the intermediate lifetime region and the recovery of spatially confined lifetime domains associated with each emitter. In phasor space, this corresponds to a pronounced compaction of the phasor distribution. Quantitatively, this reduction is captured by a strong decrease in phasor cluster area, expressed as an ellipse area ratio (*A*_after_/*A*_before_) of 1.15 × 10^–9^, together with a minimal centroid displacement of 0.0045 in (G,S) units, indicating effective suppression of TB while preserving the intrinsic decay signatures.

For comparison, the same simulated data set was processed using Richardson-Lucy (RL) deconvolution prior to phasor analysis (Figure 8c). The Richardson-Lucy method was performed using the in-built function from the skimage Python library, a gaussian PSF with standard deviation of 5 pixels and kernel size of 2 was utilized for the deconvolution process, and it was executed with 43 iterations. Compared with the MSSR-based workflow, both intensity and modulation lifetime images exhibit no separation of the fluorophore signals at these conditions. The modulation lifetime map also depicts a continuous transition between the two lifetime populations. Consistently, the corresponding phasor distribution retained a continuous bridge of intermediate phasor populations connecting the end point clusters, indicating that overlap-dominated pixels remained after the deconvolution and masking procedure under the conditions evaluated. Quantitatively, the centroid displacement remained below the acceptance criterion established in this work (0.013), indicating preservation of the end point populations. In contrast, the resulting ellipse area ratio was 100 times smaller than that of MSSR (6.48 × 10^–11^ ) indicating a collapsed distribution, reflecting a strong reduction in statistical dispersion while intermediate phasor populations remained visibly connected.

### Mean-Shift Super-Resolution Addresses Spatiotemporal Resolution in Three-Component FLIM Images

In the preceding sections, we demonstrated that masking FLIM images with MSSR-enhanced intensity maps mitigates temporal blending arising from the spatial overlap of two independent fluorophore PSFs. Here, we extend this approach to a more complex three-component system and show that the same strategy reduces lifetime mixing originating from the spatial overlap of three distinct fluorescent species.

Figure 9 presents a three-component TD-FLIM image of a single U2OS cell labeled with DAPI (orange-brown), AF555 (green), and AF532 (pink), staining the nucleus, microtubules, and mitochondria, respectively (same sample as in Figures 1,2, and 4, but acquired using a different instrument and imaging configuration, see Methods). Figure 9a shows the diffraction-limited FLIM image of the whole cell prior to MSSR masking. The white square delineates a region of interest (ROI) containing pixels where the three labeled subcellular structures spatially overlap. The corresponding whole-cell phasor plot is presented for reference and exhibits a characteristic triangular distribution, indicative of a three-component lifetime system. Each vertex of the triangle corresponds to the intrinsic lifetime of one fluorophore: the left vertex to AF532 (longest lifetime), the central vertex to AF555, and the right vertex to DAPI (shortest lifetime). The pseudocolor encoding of the lifetime image reflects the cursor-based segmentation defined in the phasor plot.

**Figure 9:**
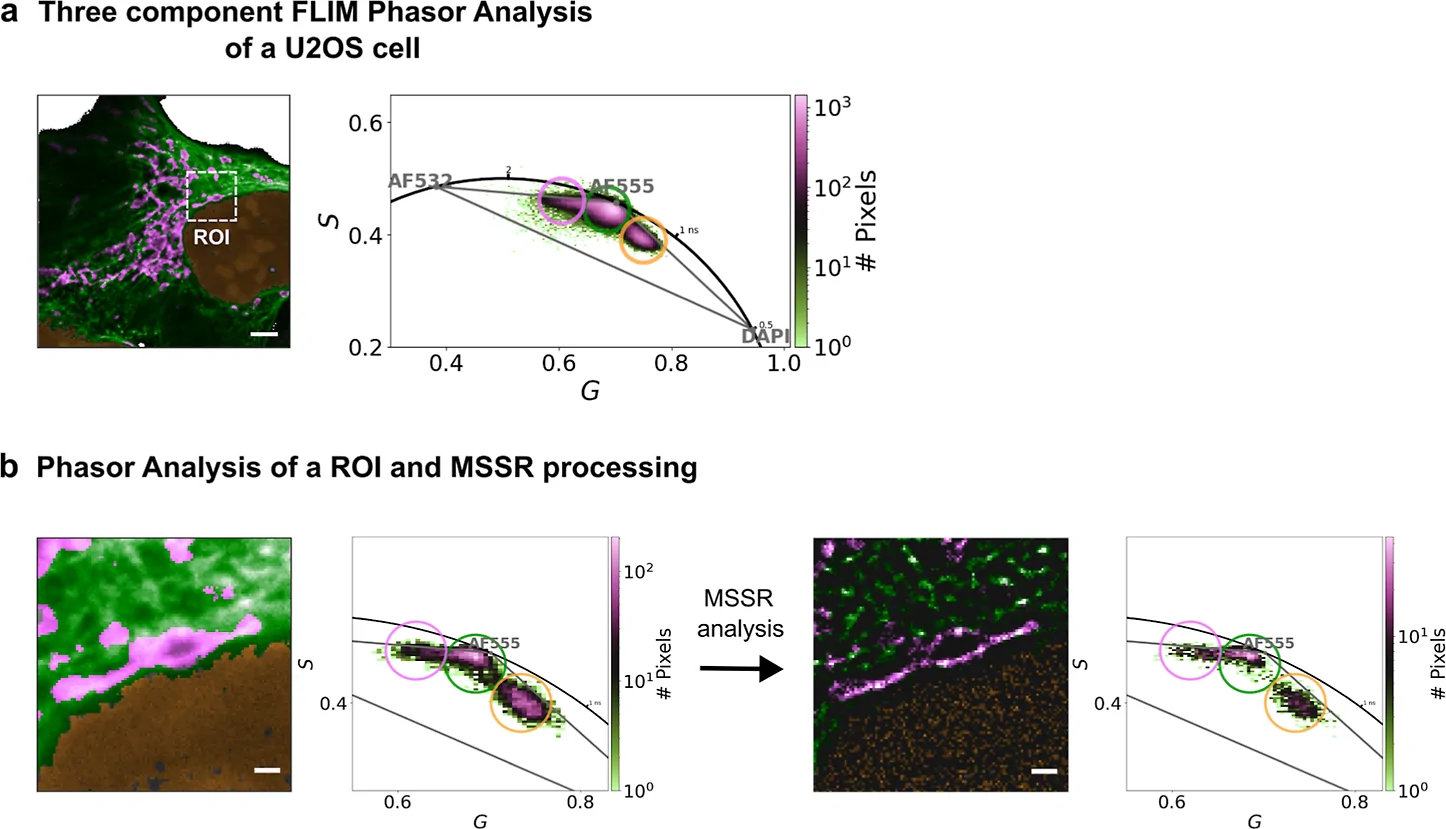
Mitigating temporal blending using mean-shift super-resolution (MSSR) on a three-component FLIM image. (a) Three-component FLIM phasor analysis of a single U2OS cell labeled with DAPI (orange-brown, nucleus), AF555 (green, microtubules), and AF532 (pink, mitochondria). Left: diffraction-limited pseudo-color FLIM image of the whole cell with the ROI indicated (white square). Right: whole-cell phasor plot showing a triangular distribution characteristic of a three-component lifetime system, with vertices corresponding to AF532 (left; longest lifetime), AF555 (center), and DAPI (right; shortest lifetime). Pseudo-color encoding reflects the cursor-based segmentation defined in the phasor plot. (b) ROI phasor analysis and MSSR processing. Left: diffraction-limited ROI image and the corresponding ROI phasor distribution (pre-MSSR). Middle: MSSR analysis step. Right: MSSR-masked ROI image using the 8% intensity threshold and the corresponding ROI phasor distribution computed after masking (post-MSSR). MSSR reduces mixed pixels while preserving phasor cluster positions. Scale bar in whole cell: 4 μm; scale bar in ROI: 800 nm.

Figure 9b presents the ROI-level analysis used to evaluate the MSSR masking. The left side of Figure 9b shows the diffraction-limited ROI (image) and its corresponding ROI phasor distribution (pre-MSSR), where mixed pixels populate the interior and edges of the triangular manifold expected for a three-component system. The right side of Figure 9b shows the same ROI after MSSR masking using an 8% intensity threshold, together with the corresponding ROI phasor distribution computed after masking (post-MSSR). Under these conditions, MSSR reduces the spatial overlap between fluorophore signals, thereby enhancing spatial separation in the ROI FLIM image. The centroids of the three phasor clusters remain stable, as confirmed by the centroid displacement metric (0.034), which remains below the predefined tolerance of 0.05 and, therefore, indicates no significant alteration of intrinsic fluorescence decay dynamics. Consistently, a modest reduction in the phasor ellipse area ratio (0.96) is observed after MSSR processing, indicating a small global decrease in the lifetime mixing within the ROI phasor distribution. Notably, the segment connecting the DAPI and AF555 phasor populations shows a pronounced depletion of intermediate pixels, yielding two more distinct clusters and indicating the suppression of temporal blending along this axis.

These findings establish that mean-shift super-resolution enables spatial refinement of FLIM images without perturbing the underlying lifetime information, even in complex, multi-component systems. The persistence of stable phasor centroids alongside reduced lifetime mixing supports a spatial—rather than temporal—origin of Temporal Blending.

## Discussion

Temporal blending in FLIM has been approached from multiple theoretical and computational perspectives, yet a unified interpretation that cleanly separates spatial from temporal contributions remains incomplete. Here, we frame TB primarily as a PSF-mediated optical mixing effect and demonstrate that applying MSSR-derived spatial masks reduces this mixing while preserving lifetime information on the retained pixels. This interpretation is supported by phasor-cluster centroid stability accompanied by a systematic reduction in the phasor cluster area.

The impact of spatial overlap on lifetime measurements was recognized in early FLIM studies. To the best of our knowledge, the first explicit description of this phenomenon was provided by Anthony Squire and Philippe Bastiaens, who introduced the term temporal blurring.^18^ Although this terminology appropriately conveys the decrease of measurement precision in FLIM, in the present work we adopt the term temporal blending to reflect more precisely the underlying mechanism: the mixing of photon streams from spatially overlapping PSFs prior to lifetime transformation. In this framework, the measured decay corresponds to a spatially weighted superposition of multiple fluorescence decays within the diffraction-limited observation volume. Importantly, TB can mimic genuine lifetime heterogeneity arising from environmental variations, molecular interactions, or conformational dynamics, thereby complicating the interpretation of the FLIM measurements.^18,19^

It is essential to distinguish this effect from fundamentally different phenomena such as diffraction in time.^55^ However, PSF-induced temporal blending represents a classical, instrumentally induced effect arising from optical diffraction, diffraction in time is a quantum-mechanical phenomenon associated with the time–energy uncertainty principle and describes the transient behavior of massive particles governed by the Schrödinger equation when encountering temporal boundaries.^55^ Despite the similarity in terminology, this effect is unrelated to the optical measurement process considered here.

Early deconvolution-based strategies, such as the Tikhonov–Miller reconstruction introduced by Squire and Bastiaens,^18^ sought to mitigate spatial blurring by applying regularization independently to each Fourier component (mean, real, and imaginary frames) to deblur FLIM images by minimizing a Tikhonov functional.^49^ While effective at sharpening images, regularization performed directly on time-varying components can differentially reshape harmonics under low signal-to-noise ratio (SNR) and/or elevated background, which may distort phasor geometry and bias lifetime estimation.

A complementary analytical framework was introduced by Yuan et al.^54^ through the FMCE, which describes how, in FD-FLIM, multiemitter measurements may become dominated by the strongest emitter. In that model, amplitude-dependent capture biases the measured lifetime toward the brightest fluorophore, producing smoothly varying lifetime distributions and spatial mislocalization artifacts. To mitigate this effect, Yuan et al. proposed a hybrid strategy in which spatial resolution is enhanced using super-resolution radial fluctuations (SRRF) on an intensity image and subsequently combined with the lifetime map. Although powerful, this approach typically relies on multiframe reconstruction and temporal interpolation. Additionally, it can be computationally demanding, because SRRF benefits from large frame counts for optimal performance.

Unlike this strategy, the present work does not reconstruct additional temporal information or modify the lifetime-estimation procedure. Instead, MSSR is applied exclusively to the intensity (DC) image prior to phasor transformation, where it generates an intensity-derived spatial discrimination mask that emphasizes emission maxima while suppressing low-intensity neighborhoods, where PSF overlap contributes to signal mixing. Phasor coordinates are subsequently computed from the retained pixel set without temporal interpolation, resampling, or manipulation of the fluorescence decay information. Consequently, MSSR does not alter the fluorescence decay associated with individual retained pixels; instead, it modifies the subset of pixels contributing to the phasor distribution by preferentially excluding the overlap-dominated regions. Therefore, the global phasor distribution is not expected to be identical before and after the masking. Rather, the expected outcome is the preservation of the end point phasor coordinates together with a reduction in the relative weighting of intermediate mixed-pixel populations, as spatially induced signal mixing is mitigated upstream of the phasor transformation.

Analysis of both two- and three-component FLIM shows that phasor-cluster centroids remain practically invariant before and after MSSR masking, supporting the preservation of the intrinsic lifetime information. Simultaneously, the marked reduction in phasor-cluster dispersion and the improved separation between components indicate that the MSSR suppresses overlap-driven mixing without introducing synthetic decay components. This behavior distinguishes MSSR from approaches that act directly on time-resolved data or implicitly modify the temporal content through reconstruction. Notably, in the most challenging three-component system, MSSR enhances the separation of AF555 and DAPI populations, reducing the extensive mixing observed in diffraction-limited FLIM and yielding more symmetric, well-resolved phasor distributions.

The experimental systems investigated in this study were deliberately selected as controlled proof-of-concept specimens since immobilized fluorophores associated with distinct subcellular structures allow PSF-mediated optical mixing to be evaluated independently of biochemical interactions. Extension of the proposed workflow to more complex biological settings, including live-cell imaging and heterogeneous specimens, is expected to introduce additional sources of variability, such as sample motion, photobleaching, dynamic changes in fluorophore brightness, heterogeneous labeling density, and molecular interactions, all of which may influence the interpretation of MSSR-masked phasor distributions. Nevertheless, the method is expected to remain most effective in applications where the primary objective is to reduce spatially induced signal mixing between adjacent structures, exhibiting distinguishable lifetime signatures. Conversely, when lifetime heterogeneity is intrinsic to the retained pixels, MSSR-FLIM should not be interpreted as a complete unmixing strategy but rather as a complementary approach for improving the spatial specificity of phasor-based lifetime analysis.

From a practical standpoint, the MSSR-derived masking strategy inevitably introduces a trade-off between the spatial discrimination and signal retention. By preferentially excluding low-probability pixels, the mask reduces the contribution of overlap-dominated regions to the phasor distribution, but it may also remove weak fluorescence structures, particularly in photon-limited data sets or specimens with low labeling density. This limitation should be considered in the broader context of FLIM, where lifetime estimation is fundamentally constrained by photon statistics, irrespective of the analysis method employed (1-3). Consequently, although MSSR has been shown to remain robust under low signal-to-noise conditions for image reconstruction,^50^ overly stringent masking may reduce the number of pixels available for phasor analysis and thus diminish the representation of low-intensity structures.

The threshold selection should therefore balance the reduction of overlap-induced mixing against the preservation of a sufficient signal for reliable lifetime estimation. Rather than relying on a single threshold value, we recommend evaluating threshold sensitivity by jointly monitoring the retained pixel fraction, the stability of the end point phasor populations, the reduction of intermediate mixed-pixel contributions, and the structural consistency of the reconstructed image. Such an empirical evaluation allows the masking level to be adapted to the characteristics of each data set while preserving the benefits of MSSR without unnecessarily compromising biologically relevant low-intensity features.

Additionally, since the MSSR-derived mask is constructed exclusively from the intensity image, its performance is inherently influenced by factors that affect fluorescence intensity, including fluorophore brightness, labeling density, excitation or detection inhomogeneity, optical attenuation, background signal, photobleaching, and, naturally, true variations in fluorophore abundance. Consequently, these factors may influence which pixels are retained for phasor analysis and, therefore, modify the statistical representation of lifetime populations within the final phasor distribution. This behavior should not be interpreted as a modification of the underlying fluorescence decay kinetics but rather as an inherent consequence of any intensity-based selection strategy. Accordingly, MSSR-FLIM is intended to reduce PSF-mediated signal mixing and improve the spatial specificity of lifetime analysis rather than to provide an unbiased quantification of fluorophore abundance without appropriate experimental controls.

The present approach is specifically designed to mitigate PSF-mediated spatial mixing arising from the diffraction-limited overlap of the neighboring fluorescence sources. Consequently, the observed improvement in lifetime discrimination originates from reducing the contribution of overlap-dominated pixels to the phasor analysis rather than from separating the intrinsically mixed fluorescence decays. Pixels that genuinely contain multiple lifetime components, *e.g*., because distinct fluorophores coexist within the same diffraction-limited region or because unresolved subpixel lifetime heterogeneity is present, remain represented by their corresponding composite decay after masking. In other words, the MSSR-FLIM approach proposed in this work was not evaluated as a method for decomposing multicomponent fluorescence decays within a single pixel. Nevertheless, dedicated phasor methodologies based on higher-order harmonic analysis have been developed for this purpose,^56^ highlighting that these approaches and MSSR-FLIM address complementary aspects of lifetime analysis, the former decomposes multicomponent fluorescence decays within individual pixels, whereas the latter mitigates PSF-mediated spatial mixing prior to phasor analysis.

The in silico characterization further shows that TB persists even when fluorophores are spatially resolvable under conventional diffraction-based criteria. Significant phasor mixing remains detectable at emitter separations as large as 4σ, intensifies near the 2σ Sparrow limit, and reaches a maximum at intermediate separations around 1.6σ under the simulated conditions. At complete overlap (0σ), the phasor distribution collapses toward the intensity-weighted linearly-combined phasor of the overlapping lifetimes. These effects emerge despite the presence of strictly non-interacting emitters, having monoexponential relaxation dynamics demonstrating that apparent lifetime heterogeneity can arise purely from PSF-mediated spatial blending.

The Richardson-Lucy comparison highlights that reducing the statistical dispersion of the phasor distribution does not necessarily eliminate intermediate phasor populations. Although the centroid displacement remained within the acceptance criterion, MSSR-FLIM performed better since Richardson-Lucy provided a 2.8 times larger centroid displacement. On the other hand, although the ellipse area became markedly more compact, a continuous bridge between the end point populations persisted in phasor space. These observations suggest that phasor compactness and the continuity of intermediate populations describe complementary aspects of the distribution and should therefore be interpreted independently. In summary, under the conditions presented in this work, Richardson–Lucy primarily reduces the statistical dispersion of the phasor distribution, whereas MSSR-FLIM preferentially reduces the continuity of intermediate phasor populations associated with PSF-mediated optical mixing.

To provide additional insight into how MSSR masking influences the relative weighting of lifetime populations in phasor space, we performed a fractional contribution analysis of lifetime components, shown in Figure S5 for the two-color FLIM case. Conventionally, fractional contributions are interpreted as the relative amplitudes or pre-exponential factors from the fluorescence decay components,^57,58^ describing the relative contribution of each component to the detected signal. Before masking, the distribution exhibits a broad transition between the two reference fluorophore populations, with a pronounced shoulder near equal fractional contributions (∼0.5). As the MSSR masking threshold increases, this intermediate shoulder progressively decreases, while the transition between the two populations becomes steeper, yielding a more negative slope, for instance, a 4% threshold yields approximately two-fold decrease in the steepness of the transition suggesting a better separation of phasor lobules; thus, enriching pixel populations associated with the dominant lifetime components (see Table S3, Supporting Information). Together, these observations are consistent with a reduced representation of pixels containing mixed fluorescence contributions, which are expected to arise predominantly from spatial overlap within the diffraction-limited image.

Importantly, this redistribution should not be interpreted as a modification of the fluorescence decay kinetics of the retained pixels. Instead, it reflects a redistribution of the relative weightings of the pixel populations contributing to the phasor analysis. Likewise, the increased representation of pixels with high fractional contributions near the 0.75 region of the plot is consistent with the preferential retention of pixels that are more likely to belong to the corresponding signal source after thresholding. Consequently, the masking strategy inherently represents a trade-off between reducing overlap-dominated pixels and excluding low-probability pixels, particularly under photon-limited conditions. Although the observed redistribution may also reflect the influence of temporal blending on the fractional contribution distributions, a quantitative relationship between these distributions and the degree of signal overlap remains to be established. Developing such a theoretical framework could provide a physically motivated criterion for selecting the masking threshold according to the degree of the overlap. However, the development of this theoretical description is beyond the scope of the present work.

It is important to emphasize that this scenario represents a controlled boundary condition in which the geometric–optical contribution to TB is isolated through idealized assumptions. In practical biological systems, fluorophores within a diffraction-limited volume may undergo interactions such as Förster Resonance Energy Transfer (FRET), where intermolecular distance plays a critical role. However, the absence of characteristic FRET signatures^27^ in the phasor plots supports the conclusion that such interactions do not significantly contribute under the conditions examined here. More generally, the separation thresholds at which TB becomes significant will depend on multiple experimental factors, including signal-to-noise ratio, background signal, relative emitter brightness, sampling conditions, and the specific PSF model.

It is instructive to compare this approach with super-resolution strategies based on higher-order correlations, such as antibunching-based microscopy introduced by Schwartz and Oron.^59^ In that framework, spatial resolution is enhanced by exploiting the nonclassical photon statistics of fluorescence emission, effectively narrowing the PSF via higher-order intensity correlations. While conceptually distinct, both approaches highlight the central role of spatial statistics in overcoming diffraction-induced averaging. However, antibunching-based methods require photon-number-resolving detection and high photon fluxes, whereas MSSR operates within the classical intensity domain and can be readily applied to standard FLIM data sets without specialized hardware or acquisition schemes.

While MSSR mitigates temporal blending by increasing spatial separability in the intensity domain prior to lifetime estimation, complementary strategies have been developed to address statistical uncertainty in phasor space. Methods such as pawFLIM^17^ and its generalized extension, Binlets,^60^ apply a translational-invariant Haar-wavelet transform directly on the phasor images. Unlike MSSR, which sharpens spatial structures to prevent optical mixing, pawFLIM acts as an adaptive spatial binning tool that sacrifices spatial resolution in homogeneous regions to dramatically improve molecular precision under low-photon conditions, guided by the explicit covariance (uncertainty) of each phasor. A combined strategy in which MSSR is applied upstream to reduce PSF-induced mixing, followed by adaptive denoising in phasor space, could provide a unified framework that simultaneously addresses spatial overlap and overcomes shot-noise limitations.

Based on these results, we propose a mechanistic interpretation in which TB arises from PSF-weighted spatial averaging imposed by the imaging system prior to lifetime estimation. When neighboring emitters fall within overlapping diffraction volumes, their photon contributions are aggregated at the pixel level, yielding apparent decays that reflect spatially weighted superposition rather than a true molecular lifetime distribution at a single location. The persistence of phasor mixing at separations up to 4σ from the in silico characterization supports this geometric–optical origin and motivates mitigation strategies that reduce effective spatial averaging before the lifetime transform. While this interpretation is supported by the present simulations and experimental observations, further validation across diverse fluorophore systems, imaging modalities, and noise regimes will be required to establish its generality.

## Conclusions

This study identifies and addresses a fundamental limitation in FLIM, namely, the intrinsic coupling between spatial resolution and temporal accuracy caused by temporal blending. Through combined in silico modeling and experimental validation, we demonstrate that this effect is driven by optical-PSF-mediated spatial averaging, which produces apparent lifetime mixing even for emitters showing strictly monoexponential fluorescence decay.

By applying mean-shift super-resolution (MSSR) as a spatial pre-processing step prior to phasor transformation, we show that TB can be substantially mitigated without introducing lifetime artifacts. MSSR acts as a probabilistic spatial filter operating exclusively on intensity information, thereby preserving the intrinsic fluorescence decay kinetics. Phasor analysis confirms this preservation through practically invariant centroid positions and reduced phasor cluster spread corresponding to reduced temporal mixing. In contrast to iterative deconvolution or temporally interpolative super-resolution approaches, MSSR enhances spatial resolution without distorting the phasor geometry or decay information. Another strength of MSSR is its capability of enhancing spatial resolution within one frame of microscopy images, thus significantly lowering the computational resources required to process the data.

Overall, the MSSR-FLIM framework offers a conceptually robust and computationally efficient strategy for improving spatial resolution while preserving temporal fidelity. These results also highlight the phasor approach as an effective tool to diagnose and quantify the influence of spatial filtering on lifetime data. The methodology is immediately applicable to live-cell FLIM, super-resolution workflows, and lifetime unmixing, offering a generalizable strategy to overcome spatial–temporal trade-offs in modern fluorescence microscopy.

## Methods and Materials

All computational analyses were implemented as a modular, notebook-based workflow in the public MSSR-FLIM-2025 repository hosted on GitHub. The repository code/directory contains notebooks encoding distinct processing stages, including instrument export handling, phasor computation and visualization, cursor-based segmentation, MSSR-based spatial masking, synthetic simulations, and downstream quantification. The present section is structured to mirror this modular organization with each methodological block defined independently and extended where required.

Repository: https://github.com/MarioGoG/MSSR-FLIM-2025

### Data Set, Specimen Preparation, and Labeling Strategy

Primary experimental analyses were performed on time-domain confocal FLIM data acquired from a standardized multicolor FLIM slide containing fixed human osteosarcoma U2OS cells (GATTA-Cells 3C FLIM; GATTAquant). The sample comprises three spectrally distinct labels: nuclear DNA labeled with DAPI, mitochondria labeled by TOM20 immunolabeling with Alexa Fluor 532, and microtubules labeled by α-tubulin immunolabeling with Alexa Fluor 555. Samples were mounted in ProLong Diamond antifade medium and sealed.

The immunolabeling strategy anchors fluorophores to distinct macromolecular structures, supporting a working model of spatially interlaced yet non-interacting fluorescent species at the scale of labeled structures. The same slide was used for two-color (Alexa Fluor 532/Alexa Fluor 555) and three-color (including DAPI) FLIM experiments. All channels were processed independently by using an identical phasor-analysis framework.

### FLIM Image Acquisition

Time-resolved confocal FLIM images were acquired on a custom microscope platform (iRT-FLIM-001; Intek Scientific). Excitation was provided by a 515 nm pulsed laser (Prima; PicoQuant) operating at a 25 MHz repetition rate. Images were collected using a 60× objective (UPLXAPO60; Olympus), a 0.5 Airy unit pinhole, and a pixel size of 54 nm. Emission was filtered to 550–614 nm using a bandpass filter (FF01-582/64-25; Semrock) and detected with a photomultiplier tube (H10720-01; Hamamatsu Photonics). For each pixel, the acquisition yielded a time-domain fluorescence decay sampled over a fixed nanosecond-scale temporal window. A modulation of 50 MHz (2nd harmonic) was used for the calculation of G and S phasor coordinates.^61^

Selected data sets were additionally acquired on a Leica TCS SP8 STED 3X platform equipped with a FALCON-FLIM module (photon-counting hybrid detectors, galvanometric scanning, and a 63× high-numerical-aperture oil-immersion objective). The acquisition frequency for this system was 78 MHz. These data sets were analyzed with the same conceptual workflow (phasor-based segmentation with optional MSSR-derived spatial masking); instrument-specific acquisition parameters are detailed in the corresponding subsection.

### Phasor Computation and Data Handling

Phasor analysis followed the standard FLIM phasor formalism and the Weber identities. Time-domain decays were converted to per-pixel phasor coordinates (G, S) and mean intensity (DC component). Dual modulation frequencies (50 and 100 MHz) were implemented via cosine and sine transforms and accelerated on CUDA-enabled GPUs when available (LifetimeXplorer v1.10.9). For downstream Python-based processing, DC images were imported from TIFF files (e.g., *_mean.tif) and (G,S) matrices from CSV (e.g., *_real.csv, *_imag.csv) using tifffile and pandas, respectively.

For the Leica system, phasor analysis was performed using the manufacturer’s built-in software with a modulation frequency of 80 MHz. The raw time-correlated single-photon counting (TCSPC) data were exported in PicoQuant’s proprietary PTU file format. The results of the phasor analysis were exported as a Leica Image File (.lif), a proprietary Leica file format containing the real (G) and imaginary (S) phasor coordinates.

### Frequency Conventions, Harmonic Support, and Calibration

Downstream phasor operations followed explicit frequency parametrization PhasorPy conventions. A fundamental frequency of 50 MHz (0.05 GHz) was used. Harmonic support was enabled by specifying a harmonic index (e.g., harmonic = 1 for the fundamental). When reference-based calibration was required, calibration utilities (e.g., phasor_calibrate) were applied by using a reference lifetime-derived phasor and a measured reference to correct systematic offsets.

### Intensity Thresholding and Spatial Stabilization of Phasor Maps

To suppress noise-driven phasor scatter, low-count pixels were excluded before visualization, segmentation, and lifetime extraction. Thresholding was applied to the DC image to generate a binary support mask that removed background and low-count regions. Unless otherwise stated, (G,S) and derived lifetime quantities were computed from the original decay data. Representative preprocessing parameters included a minimum intensity threshold (mean_min = 7) and optional Gaussian smoothing (scipy.ndimage.gaussian_filter, sigma = 1 pixel). To reduce salt-and-pepper noise in the phasor coordinate maps while preserving structural boundaries, repeated median filtering with intensity weighting was applied by the use of the in-built PhasoPy’s function phasor_filter_median­(image, G, S; size = 3, repeat = 3) corresponding to a 3 × 3 spatial median filter iterated three times and weighted by the smoothed mean intensity.

### Phasor Visualization and Reciprocity Mapping

Phasor distributions were rendered as density plots in (*G*, *S*) space with a universal semicircle. Monoexponential decays lie on the semicircle, whereas multiexponential mixtures fall inside the semicircle due to an amplitude-weighted linear combination. Operational reciprocity mapping was used bidirectionally: pixels selected by a phasor-space region were mapped back to image space to generate pseudo-color masks, and the inverse mapping was used to validate that spatially coherent structures corresponded to coherent phasor regions. Representative visualization settings included 2D histogram rendering with bins = 150 and axis limits tuned to the informative region with representative values being G = (0.55 to 1.00) and S = (0.00 to 0.60).

### Cursor-Based Segmentation and Pseudo-Color Classification

Segmentation in the phasor space used circular cursors. For a cursor *k* centered at (*G*_*k*_, *S*_*k*_) with radius *r*_*k*_, pixel membership was defined using the built-in cursor function from PhasorPy.

For two-component analyses, cursors were placed at end point clusters corresponding to dominant fluorophore populations. For three-component data sets, an additional cursor was positioned over the intermediate region connecting end points to capture mixed pixels consistent with amplitude-weighted linear combinations. Representative full-field end point cursor centers were near (*G*_1_, *S*_1_) = (0.705,0.445) and (*G*_2_, *S*_2_) = (0.81, 0.393), with radii on the order of 0.04.

### ROI-Based Local Subanalyses

Local analyses were performed by defining regions of interest (ROIs) via array slicing and applying the same pipeline (thresholding, optional median filtering, phasor mapping, segmentation, pseudocolor mapping, and histogram-based quantification). Representative ROI definitions included ROI1, ROI2, ..., and ROIn.

Cursor geometries and placements were held constant within unmasked versus masked comparisons to isolate the impact of spatial masking on the phasor dispersion. Pseudocolor overlays were rendered by alpha blending categorical labels onto a grayscale mean-intensity background derived from the thresholded mean image. Spatial scaling used 54 nm per pixel for the scale bars and overlays.

### Apparent Lifetime Extraction from Phasor Coordinates

Apparent lifetimes were computed per-pixel from (*G*,*S*) at modulation frequency *f* with ω = 2π*f*. Phase and modulation were computed as

1 $$\phi =\arctan\left(\frac{S}{G}\right)$$

2 $$M=\sqrt{G^{2}+S^{2}}$$

Phase lifetime (τ_ϕ_) and modulation lifetime (τ_*M*_) were computed via Weber identities

3 $$\tau_{\phi }=\frac{1}{\omega }\cdot \frac{S}{G}$$

4 $$\tau_{M}=\frac{1}{\omega }\cdot \sqrt{\frac{1}{\left(G^{2}+S^{2}\right)}-1}$$

For monoexponential decays, phase and modulation lifetimes coincide.

Lifetime extraction was performed after intensity thresholding (and after median filtering when enabled) with emphasis on modulation lifetime distributions.

### Apparent Lifetime Histograms and Gaussian-Mixture Fitting

Lifetime distributions were analyzed using histograms of apparent modulation lifetimes τ_*M*_ over end point-enriched pixels or over end point plus intermediate TB pixels, depending on the analysis objective. Two fitting regimes were used: (i) end point-only analyses with representative settings bins ≈95 and range ≈ (0.6 to 2.6) , fitted with a two-Gaussian mixture; and (ii) end point plus intermediate analyses with representative settings bins ≈200 and range ≈ (0.6 to 2.6), fitted with a three-Gaussian mixture. Gaussian mixtures were fitted using scipy.optimize.curve_fit with parametric amplitude, mean and standard deviation components. The user can choose physically plausible bounds on component means and widths. Fit initializations were selected from visually apparent histogram modes and recorded per ROI.

### Quantification of MSSR Effects

Two complementary metrics were used to evaluate whether MSSR-based masking altered lifetime signatures or primarily reduced the dispersion.

Centroid displacement (phasor stability) was computed for each end point cluster as the Euclidean distance between (*G*,*S*) centroids before and after masking. To compute the phasor cluster centroids we used

5 $$\left(\mathrm{G̅},\mathrm{S̅}\right)=\left(\frac{1}{N}\sum_{i=1}^{N}G_{i},\frac{1}{N}\sum_{i=1}^{N}S_{i}\right)$$

So that the Centroid displacement was calculated as

6 $$\Delta_{r}=\sqrt{{\left({\mathrm{G̅}}_{after}-{\mathrm{G̅}}_{before}\right)}^{2}+{\left({\mathrm{S̅}}_{after}-{\mathrm{S̅}}_{before}\right)}^{2}}$$

Displacements were interpreted relative to a conservative no distortion bound of 0.05 phasor units, selected to exceed expected fluctuations from photon noise and finite sampling.

Cluster compactness (dispersion reduction) was summarized by using confidence-ellipse area estimates. Compactness was quantified as an ellipse area ratio

7 $$R_{A}=\frac{A_{after}}{A_{before}}$$

The ellipses areas were computed with the following equations, considering the estimation of an area with 95% confidence interval

8 A=abπ

9 $$a=\sqrt{\lambda_{1}{\chi_{\alpha }}^{2}}$$

10 $$b=\sqrt{\lambda_{2}{\chi_{\alpha }}^{2}}$$

Where the parameters λ_1_,λ_2_ are the eigenvalues from the covariance matrix that quantify the variance along the principal axes of the phasor distribution, being λ_1_≥λ_2_. Thus, determining the lengths of the semi-axes of the phasor cluster by scaling them by the 95% confidence factors χ_α_^2^ = χ_0.95 , 2_^2^ = 5.991.

Values below unity indicate a reduced dispersion. This metric was evaluated for whole-cell distributions and for ROIs to quantify local suppression of dispersion under stable end point coordinates. Together, these metrics distinguish shifts in lifetime-coordinate end points, indicative of altered decay signatures, and evaluate reduced dispersion at stable end points, consistent with mitigated mixing while conserving end point lifetimes.

### Mean-Shift Super-Resolution as a Masking Strategy for Spatial Separability

Mean-shift super-resolution (MSSR) was used as an intensity-domain spatial separability enhancer to generate spatial masks while preserving time-resolved decay information. MSSR follows local intensity gradients (mean-shift procedure) to concentrate probability density toward local maxima, thereby reducing effective point-spread function overlap and improving separability of nearby structures without direct manipulation of the time-resolved decay.

The intermediate MSSR output ( $P_{MSSR^{n}}\in \left[0,1\right]$ ) was used to generate thresholding masks. This intermediate output was treated as a probability-like spatial map suitable for threshold-based retention of high-probability pixels while suppressing low-probability regions associated with background and overlap. Unless otherwise stated, masks were derived from the first iterative output $P_{MSSR^{1}}$ (corresponding to MSSR^1^ in the accompanying scripts). Binary masks were constructed by thresholding $P_{MSSR^{n}}$ at selected levels. Threshold selection was explored using quartile-like cutoffs (0.25, 0.50, 0.75) in simulations and then transferred to experimental data using thresholds that retained structural continuity while suppressing overlap-dominated interface regions. The resulting binary masks were applied to the precomputed (*G*,*S*) images, ensuring that lifetime estimation remained based on the original decay data. This thresholding step was performed using the thresholding function from PhasorPy library. Representative MSSR parameters used in the implemented pipeline included sigma_px = 2.43 (PSF-width estimate in pixels), order = 1 (MSSR^1^), width = 0, mesh = 0, ftI = 0, amplification = 0, and intNorm = False. A representative masking threshold applied to the $P_{MSSR^{1}}$ map in experimental analyses was mean_min = 0.04. MSSR processing used napari_superres.

### Sparrow-Limit Gaussian Emitter Simulations for MSSR-Derived Probability-Support Mapping

To illustrate how MSSR improves separability at the Sparrow criterion using the bounded intermediate output $P_{MSSR^{n}}$ , synthetic data was generated from two equal-intensity Gaussian emitters and processed in one and two spatial dimensions. Two emitters were modeled as identical Gaussian intensity distributions with standard deviation σ. In one dimension, the individual contributions were defined as

11 $$I_{L}\left(x\right)=Ae^{{\left(x+x_{0}\right)}^{2}/2\sigma^{2}}$$

for the left fluorophore, and

12 $$I_{R}\left(x\right)=Ae^{{\left(x+x_{0}\right)}^{2}/2\sigma^{2}}$$

for the right fluorophore. For the sake of simplicity the simulation was performed with equal amplitudes *A*. The composite raw signal was then computed as the sum of both distributions

13 $$I_{Raw}\left(x\right)=I_{L}\left(x\right)+I_{R}\left(x\right)$$

The emitter separation was set to the Sparrow limit for equal-width Gaussians by imposing a center-to-center distance of 2σ, implemented as *x*_0_ = σ. The horizontal axis was expressed in units of σ by plotting the dimensionless coordinate $u=\frac{x}{\sigma }$ . For visualization, profiles were normalized by their maximum value

14 $$Ĩ\left(u\right)=\frac{I\left(u\right)}{I\left(u_{\max}\right)}$$

Signals were evaluated on a uniform grid spanning [*u*_min_, *u*_max_] with *u*_min_ = −4 and *u*_max_ = 4, corresponding to [-4σ,4σ]. A fixed sampling step Δ*u* was used to ensure smooth profiles. The same axis definition was used to generate one-dimensional profiles and to define the coordinate system for the two-dimensional fields.

MSSR was applied to the normalized raw intensity field (one-dimensional or two-dimensional). Two MSSR orders were evaluated: MSSR^0^ (first-stage mean-shift transformation output) and MSSR^1^ (one additional mean shift iteration). For downstream masking, the workflow described above was used.

### Simulation Framework for TD-FLIM and FD-FLIM Signals and Phasor-Coordinate Validation

Synthetic simulations were used to validate phasor behavior for monoexponential versus multiexponential decays and to illustrate the equivalence between TD-derived and FD-derived phasors under matched modulation frequency and calibration assumptions. Simulations were implemented in Python using numpy and scipy and generated only one-dimensional temporal signals.

Global simulation settings were parameterized by modulation frequency *f* and angular frequency ω, defined as ω = 2π*f*. The modulation period is computed from the inverse of the frequency, $T=\frac{1}{f}$ . A representative FD condition used *f* = 80*MHz*, corresponding to *T* = 12.5*ns*. Signals were discretized into samples = 256 points over one period. Signal scaling used mean = 10.0 (arbitrary units) and background = 0.0. An instrument-response term was included as a narrow, impulse-like response controlled by a phase–location parameter (zero_phase) and an optional width parameter (zero_stdev); a figure-generating configuration used zero_stdev = None to produce a narrow response.

### Time-Domain Decay Synthesis used the Expression for the Monoexponential Time-Varying Intensity

15 $$I\left(t;\tau \right)=I_{0}e^{-t/\tau },\tau \in \left\{0.5,1.0,2.0,4.0\right\}ns$$

and the multiexponential decays follow the expression for amplitude-weighted mixtures

16 $$I_{multi}\left(t\right)=\sum_{i=1}^{N}a_{i}e^{-t/\tau_{i}},\sum_{i=1}^{N}a_{i}=1$$

A representative two-component mixture used equal fractions (fractions = [0.5, 0.5]), and a single-component reference decay was generated for calibration and validation.

Frequency-domain analogs were generated over one period and rendered on a phase axis ? in degrees derived from time *t* by

17 $$\phi \left(t\right)=\frac{t}{T}\times 360^{◦}$$

Composite FD signals were generated by a linear combination of component contributions consistent with the amplitude fractions used in the TD mixtures.

Phasor coordinates were computed from lifetimes using the signal-to-phasor function from the PhasorPy library. A representative reference lifetime used for calibration was ref_lifetime = 0.3 ns. Agreement was enforced by numerical tolerance checks (numpy.testing.assert_allclose).

### In Silico FLIM Model for Two Non-interacting Fluorophores with Controlled Spatial Overlap

To quantify temporal mixing under PSF overlap and to test MSSR-based mitigation under fully parametrized conditions, a two-emitter in silico model was implemented as repository-tracked notebooks. The simulation workflow used PhasorPy for FLIM signal synthesis and phasor transforms, numpy for array construction, scipy (notably scipy.signal) for convolution, and matplotlib for visualization. MSSR operations were executed through the napari_superres.

Two independent fluorophores were modeled as monoexponential emitters with lifetimes τ_1_ = 1*ns* and τ_2_ = 4*ns*. Temporal sampling was defined by a modulation frequency *f* = 80*MHz*, with ω = 2π*f* and laser period *T* = 1/*f* = 12.5*ns*. Temporal waveforms were discretized with N = 256 samples per period/binning window. The baseline intensity scale used mean = 1e4 and background = 0. A narrow, impulse-like instrument-response proxy was included by specifying zero_phase = 0.08 and zero_stdev = None, and phasor calibration used a reference lifetime of 0.06 ns.

Time-resolved fluorescence signals were synthesized using PhasorPy’s lifetime-to-signal simulator for single-component decays and a two-component mixture with equal fractional contributions (fractions = [0.5, 0.5]) to validate amplitude-weighted linearity in phasor space. Each fluorophore’s temporal signal *I*_*k*_(*t*) was assigned to a single pixel location (*i*_*k*_,*j*_*k*_), encoding temporal information at spatially discrete emitter coordinates prior to optical blending.

A synthetic FLIM image stack *F*(*y*,*x*,*t*) was constructed with spatial dimensions x_dim = 127, y_dim = 127 and temporal dimension t_dim = 256. Emitters were placed on the central row (cy = center = 63) with controlled horizontal separations expressed in units of the PSF standard deviation σ. Spatial sampling used pxsz_nm = 5 nm per pixel and an isotropic Gaussian PSF with sigma_nm = 100 nm, giving σ_*px*_ = σ_*nm*_/*pxsz*_*nm*_ = 20 pixels and $FWHM=2\sqrt{2\ln2}$ , σ_*px*_. Separation conditions included complete overlap and separations spanning the range from sub-resolution to well-resolved, including a minimum-separation condition at 1.6σ implemented as Δ = 0.8σ_*px*_, and additional separations such as 2.0σ, 2.5σ, 3.0σ, 4.0σ, and 6.0σ via Δ = {1.0,1.25,1.5,2.0,3.0}­σ_*px*_.

Optical blending was modeled by convolving each time slice with the Gaussian PSF. For each time index *t*

18 $$F_{blended}\left(\cdot ,\cdot ,t\right)=\left(F_{1}{\left(\cdot ,\cdot ,t\right)}^{*}PSF\right)+\left(F_{2}{\left(\cdot ,\cdot ,t\right)}^{*}PSF\right)$$

where * denotes 2D convolution computed using FFT-based convolution (scipy.signal.fftconvolve, mode = “same”). This produced a time-resolved image stack in which temporal information remained fluorophore-specific at the source level but became spatially mixed through PSF overlap.

Per-pixel phasor components were computed by Fourier decomposition along the temporal axis using PhasorPy’s function phasor_from_signal function. Systematic offsets were corrected by reference-based calibration (phasor_calibrate) using τ_ref_ = 0.06*ns* at *f* = 80*MHz*. Background suppression was implemented by intensity thresholding on the mean component using phasor_threshold with mean_min = 0.75, yielding thresholded maps *DC*_*th*_,*G*_*th*_,*S*_*th*_ for lifetime extraction and visualization.

Phase and modulation were computed using eq 1 and eq 2 ,respectively. To stabilize calculations in low-amplitude regimes, lower limits were imposed as mod_lower_limit = 0.36 and phase_lower_limit = 0.1 prior to lifetime mapping; multiplicative scaling factors were unity (factor_tm = 1, factor_tp = 1), and optional correction terms were disabled (mod_correction = false, phase_correction = false) to preserve correspondence with analytic phasor expressions.

For quantitative summaries, lifetimes were sampled along the central row and at three landmark pixels: the two emitter-coordinate pixels (*x*_1_, *y*_*c*_) and (*x*_2_, *y*_*c*_), and the midpoint (*x*_*c*_, *y*_*c*_) (saddle/reference pixel). These values were tracked across separations to quantify the dependence of the recovered apparent lifetimes on spatial overlap.

Phasor-space segmentation for simulated data used polar cursors defined by phase and modulation bounds, implemented with mask_from_polar_cursor. Three classes were encoded via.

- phase_min = [0.78, 0, 0.453], phase_max = [1.13, 0, 0.555]
- modulation_min = [0.438, 0, 0.77], modulation_max = [0.568, 0, 0.905]

Cursor overlays were rendered using PhasorPlot.polar_cursor, and reciprocity mapping to image space was visualized with pseudocolor, using the thresholded mean intensity as the support image.

### MSSR-Based Mitigation of Temporal Mixing in Simulated FLIM Data

MSSR processing was performed on the mean intensity projection *DC*(*y*,*x*) obtained from the phasor decomposition. MSSR was executed via napari_superres setting order = 1 (MSSR^1^), fwhm = 2.4 (PSF width parameter in the MSSR interface), and disabled auxiliary options (amp = 0, mesh = 0, ftI = 0, intNorm = 0). The MSSR-processed intensity map was used as a mask using the threshold function from PhasorPy and three levels of thresholding were computed using mean_min = {0.25, 0.5, 0.75}. Lifetime extraction (τ_*M*_,τ_ϕ_) were then repeated using the same modulation frequency (80 MHz), the same Weber identities, and the same numerical lower limits (mod_lower_limit = 0.36, phase_lower_limit = 0.1). Comparisons between pre- and post-MSSR simulations were performed at the level of mean intensity structure, phasor distributions in (G,S) space, and apparent lifetime maps.

### Three-Component TD-FLIM Phasor Analysis and MSSR Masking

A three-component cellular FLIM data set was analyzed in phasor space to evaluate MSSR-mediated suppression of Temporal Blending in regions of multi-structure spatial overlap. The specimen was collected with a Leica TCS SP8 STED 3X platform equipped with a FALCON-FLIM module (photon-counting hybrid detectors, galvanometric scanning, and a 63× high-numerical-aperture oil-immersion objective), corresponds to the same fixed-cell multicolor FLIM slide used throughout this work, including the DAPI channel for three-color TD-FLIM acquisitions; each spectral channel was processed independently using the same phasor formalism. The region of interest (ROI) reported in Figure 9 was selected in the diffraction-limited image to include spatial overlap among nucleus (DAPI), microtubules (AF555), and mitochondria (AF532), thereby maximizing the prevalence of mixed pixels for downstream evaluation.

Phasor coordinates were computed directly in the processing notebook using the PhasorPy (phasorpy) Python library. Time-tagged photon data were imported from PicoQuant.ptu files and organized as time-resolved image stacks with axes (x, y, t), where t indexes the TCSPC histogram bins over the 80 MHz excitation period. Pixel-wise phasors (G, S) and the mean intensity (DC component) were obtained by Fourier decomposition at the selected analysis harmonic using the PhasorPy implementation, yielding spatial maps of DC, G, and S and a corresponding phasor distribution in the (G, S) space.

Instrument-related phase shifts and timing offsets were corrected by reference calibration using a homogeneous lifetime standard acquired under the matched acquisition settings. A homogeneous solution of ATTO 488 (200 nM) was used as the calibration reference. The reference phasor was computed from this standard within the same PhasorPy workflow and used to calibrate experimental phasors by aligning the measured reference phasor to the theoretical phasor expected for the ATTO 488 reference lifetime at an 80 MHz modulation frequency. The resulting calibration transform was applied to the three-component cellular data set prior to phasor visualization, cursor-based segmentation, and MSSR-masked reanalysis.

To suppress background and low-count pixels, intensity thresholding was applied to the DC image to generate a binary support mask that was propagated to the G and S maps prior to visualization and segmentation. Spatial stabilization of the phasor maps was performed by median filtering of G and S using a small kernel (3 × 3) with repeated application. Three-component behavior was assessed by the emergence of a triangular phasor distribution. End point populations were identified by cursor selection near each vertex of the triangle, and reciprocity mapping was used to project phasor selections back into image space for visualization of the spatial distribution of each component and mixed regions.

For MSSR-assisted mitigation of temporal mixing, mean-shift super-resolution (MSSR^1^) was applied to the mean-intensity image to generate an MSSR-derived probability-like map. The MSSR output was rescaled to [0, 1], and a fixed threshold (8% in Figure 9) was applied to create a binary MSSR mask that restricts analysis to high-likelihood emitter regions prior to phasor transformation and segmentation. The MSSR mask was applied to the ROI data set, and phasor distributions were recomputed from the masked pixels to evaluate changes in cluster compactness and mixing signatures. Cluster compactness was quantified using an ellipse area ratio, defined as the area of a covariance-based confidence ellipse fitted to a cluster after MSSR masking divided by the area before masking. Centroid displacement was quantified as the Euclidean distance between pre- and postmasking cluster centroids in (G, S) space. These metrics were used to confirm reduced phasor dispersion (reduced mixing), together with centroid stability (preservation of intrinsic decay signatures).

### Richardson-Lucy Deconvolution

Richardson-Lucy deconvolution was performed using the built-in function from the skimage library from Python. A user function was defined to produce a Gaussian kernel (generate_gaussian_psf­(sigma, size), and the sigma parameter was defined as a quarter of the standard deviation from the Gaussian functions to produce the simulated PSFs. On the other hand, the kernel size was defined as a 2x2 Gaussian kernel.

To perform the Richardson-Lucy deconvolution, the richardson_lucy function was used importing from the skimage.restoration library. The number of iterations was set to 43, chosen as the maximum number of iterations before the ellipse ratio analysis of its phasor cluster yielded nan.

### Fractional Contribution Analysis

A fractional contribution analysis from the phasor plot was performed by using the PhasorPy library. To perform this analysis, the following libraries were imported: phasor_component_fit, phasor_component_fraction, and phasor_component_graphical from the phasor.component library. The lifetime components were defined within a list as in component_lifetimes = [1.5, 2.3], and the values used from the lifetime components were obtained from the GattaQuant webpage. To transform these lifetime signatures to phasor space, the function phasor_from_lifetime from PhasorPy was used before fractional analysis.

### Brightness and Contrast

To improve the visualization of the FLIM-MSSR analysis, the brightness and contrast of the pseudo-color FLIM images were adjusted by linearly rescaling the display intensity range prior to rendering. Brightness was controlled by shifting the center of the displayed intensity interval using a defined variable center = (new_vmin + new_vmax)/2, whereas contrast was adjusted by proportionally expanding the displayed dynamic range about this center through the user-defined variable span = (new_vmax - new_vmin) * contrast. In the images presented here, brightness and contrast scaling factors of 1.5 and 2.5, respectively, were applied uniformly to all pseudo-color images to improve the visualization of lifetime distributions. These adjustments affected only the image display and were not used for quantitative analysis or phasor calculations.

### Software, Libraries, and Computational Environment

Experimental data processing, visualization, phasor operations, filtering, MSSR masking, and model fitting were performed in Python using numpy, pandas, scipy (including scipy.ndimage and scipy.optimize), skimage, matplotlib, tifffile, PhasorPy (phasorpy), and napari_superres. Simulation plotting additionally used Cmcrameri for colormap selection. Phasor coordinate computation from time-domain decays was performed in LifetimeXplorer v1.10.9 with GPU acceleration when available, exporting mean intensity and (G, S) matrices for Python-side processing.

## Supplementary Material



## Notes

Phasor representation of TD- and FD-FLIM and phasor mixing; simulated PSF-overlap and temporal blending analysis; FD-FLIM in silico simulations; MSSR masking and threshold-dependent mitigation of temporal blending; and quantitative phasor-cloud and fractional-contribution metrics (PDF)

## Notes

Analysis scripts and notebooks used to reproduce MSSR-FLIM processing, simulations, and quantitative metrics are available in the project repository: https://github.com/MarioGoG/MSSR-FLIM-2025.git. All images analyzed are available via the Zenodo repository and can be retrieved from the DOI provided here: “Two- and Three-color confocal FLIM images of U2OS Gatta Quant samples” 10.5281/zenodo.19028067
