# Supplementary material for: PSF-Driven Spatiotemporal Blending in Fluorescence Lifetime Imaging Microscopy and Its Mitigation via Mean-Shift Super-Resolution-Based Masking
Source: ACS Photonics. 2026 Sep 14;13(19):5571–90. doi: 10.1021/acsphotonics.6c00741 (PMC13651706; doi:10.1021/acsphotonics.6c00741)
Supplement: Supplementary file 1 [file ph6c00741_si_001.pdf]

SUPPORTING INFORMATION

PSF-Driven Spatio-Temporal Blending in Fluorescence Lifetime Imaging Microscopy and Its Mitigation via Mean-Shift Super-Resolution-Based Masking.

Mario González-Gutiérrez[1], Diana M. Vázquez-Enciso[1], Nicolás Mateos[2], Wonsang Hwang[3], Esley Torres-García[4], Haydee O. Hernández[5], Jenu V. Chacko[6], Iván Coto Hernández[7], Pablo Loza-Álvarez[2], Christopher Wood[1], Adan Guerrero[1,8,*]

Affiliations:

1. Laboratorio Nacional de Microscopía Avanzada, Instituto de Biotecnología, Universidad Nacional Autónoma de México, Cuernavaca, Morelos, Mexico.
2. Institut de Ciencies Fotoniques, Castelldefels, Catalunya, Spain.
3. Wellman Center for Photomedicine, Harvard Medical School, Massachusetts General Hospital, CNY149, 13th St, Charlestown, 02129, MA, USA.
4. Facultad de Ingeniería, Universidad de la República, Montevideo, Uruguay.
5. Centro de Investigación en Ciencias, Universidad Autónoma del Estado de Morelos, Cuernavaca, Morelos, Mexico.
6. Laboratory for Optical and Computational Instrumentation, University of Wisconsin at Madison, 1675 Observatory Dr., Madison, WI 53706, USA.
7. Center for Interdisciplinary Innovation in Imaging, Massachusetts General Hospital and Harvard Medical School, 149 13th St, Charlestown, 02129, MA, USA.
8. Centro Internacional de Ciencias AC. Cuernavaca, Morelos, Mexico.

*Corresponding author: adan.guerrero@ibt.unam.mx

CONTENTS

S0. Organization of the Supporting Information..S2

S1. Comparison between Time Domain- and Frequency Domain-Fluorescence Lifetime Imaging Microscopy and the Representation of the temporal information in Phasor Space..S2

S2. Phase lifetime transition shift in the PSF-overlap regime..S4

S3. FD-FLIM in silico simulation of Temporal Blending induced by PSF overlap..S5

S4. Percentile-based MSSR masking progressively reduces Temporal Blending at the Sparrow limit..................S6

S6. Effect of MSSR masking on the phasor representation and fractional contribution of lifetime populations. S8

S7. References..S9

S0. Organization of the Supporting Information

The Supporting Information is organized into six sections to facilitate navigation. Depending on the content, each section contains supplementary figures, supplementary tables, or both. Section S6 includes both figures and tables.

Total supplementary figures: 5
Total supplementary tables: 3

S1. Comparison between Time Domain- and Frequency Domain-Fluorescence Lifetime Imaging Microscopy and the Representation of the temporal information in Phasor Space.

The core claim evaluated here is that intermediate apparent lifetimes can arise from optical mixing; accordingly, here we establish the relevant phasor geometry and the relationship between time-domain (TD) and frequency-domain (FD) FLIM using a didactic overview.

TD-FLIM registers fluorescence relaxation as a time-resolved intensity decay following pulsed excitation (Fig. S1a). In contrast, FD-FLIM derives lifetimes from the phase delay and demodulation of the emission waveform relative to sinusoidally modulated excitation (Fig. S1b). Both modalities yield equivalent phasor coordinates (G, S) for the same decay at a given harmonic, taking into account calibration and instrument-response corrections; consequently, TD- and FD-FLIM provide indistinguishable phasor plots in the ideal case (Fig. S1c).

In this representation, monoexponential decays map to single points on the universal semicircle, whereas multicomponent decays and mixed signals map inside the semicircle(1-5) (Fig. S1c). Importantly, a pixel receiving photons from distinct species of monoexponential fluorescence decay emitters yields an amplitude-weighted composite decay whose phasor lies between the endpoint phasors according to the linear combination rule. This framework defines the operational signature of Temporal Blending (TB), *i.e.* intermediate phasor positions that arise from spatial photon pooling rather than from lifetime heterogeneity due to environmental parameters or molecular interactions.

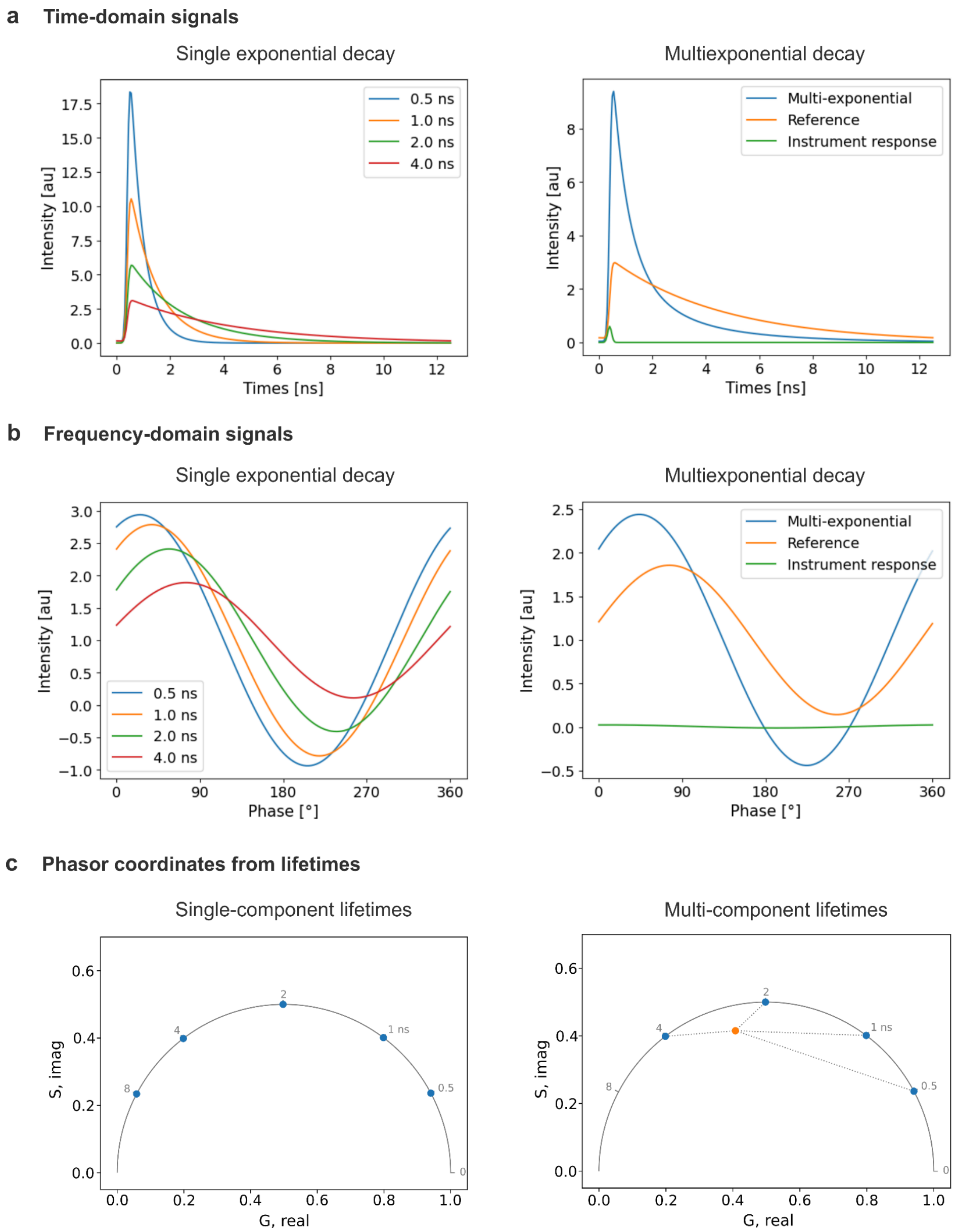

Fig. S1. Conceptual link between TD-FLIM, FD-FLIM, and phasor mixing for monoexponential versus multicomponent decays. (a) Time-domain signals. Left: Ideal monoexponential decays with lifetimes of 0.5 ns (blue), 1.0 ns (orange), 2.0 ns (green), and 4.0 ns (red). Right: A representative multicomponent (composite) decay (blue) shown alongside a reference decay used for calibration (orange) and the instrument response function, IRF (green). (b) Frequency-domain signals. Left: Frequency-domain emission waveforms for the same monoexponential lifetimes, using the same color code as in (a). Increasing the lifetime produces increased phase delay and reduced modulation. Right: Frequency-domain representation of a mixed (multicomponent) signal (blue), shown alongside the reference waveform (orange) and IRF-related contribution (green). (c) Phasor coordinates derived from the signals in (a) and (b). For a given analysis harmonic (and after appropriate calibration/IRF handling), TD-FLIM and FD-FLIM yield equivalent phasor coordinates (G, S), and therefore provide indistinguishable phasor plots in the ideal case. Left: Monoexponential lifetimes map to single points on the semicircle (labels indicate lifetime ordering). Right: A multicomponent decay maps inside the semicircle (orange point), consistent with a linear combination of lifetime components. Colors in panels (a,b) index lifetime components, whereas the orange color in panel (c, right) denotes the multicomponent phasor. Dotted segments are geometric guides illustrating that the mixed phasor lies within the convex region (interior of semicircle) spanned by the contributing monoexponential phasors; fractional contributions are encoded by the interior point position rather than by the segment lengths.

S2. Phase lifetime transition shift in the PSF-overlap regime

When two fluorophores reside within a diffraction-limited observation volume, the signals coming from each emitter mix, resulting in the linear combination of both lifetime signatures. The intermediate phasors produced by this optical mixing correspond to the Temporal Blending effect.

From our simulated model, it was observed that the smooth transition between the two fluorophores shifts towards the fluorophore having the longer fluorescence lifetime, an effect resembling the FMCE model from Yuan et al (6). It is interesting to note that this shift is more pronounced in the phase domain rather than in the modulation domain. As far as we know, however, further theoretical frameworks would be beneficial to provide a consolidated explanation of this phenomenon.

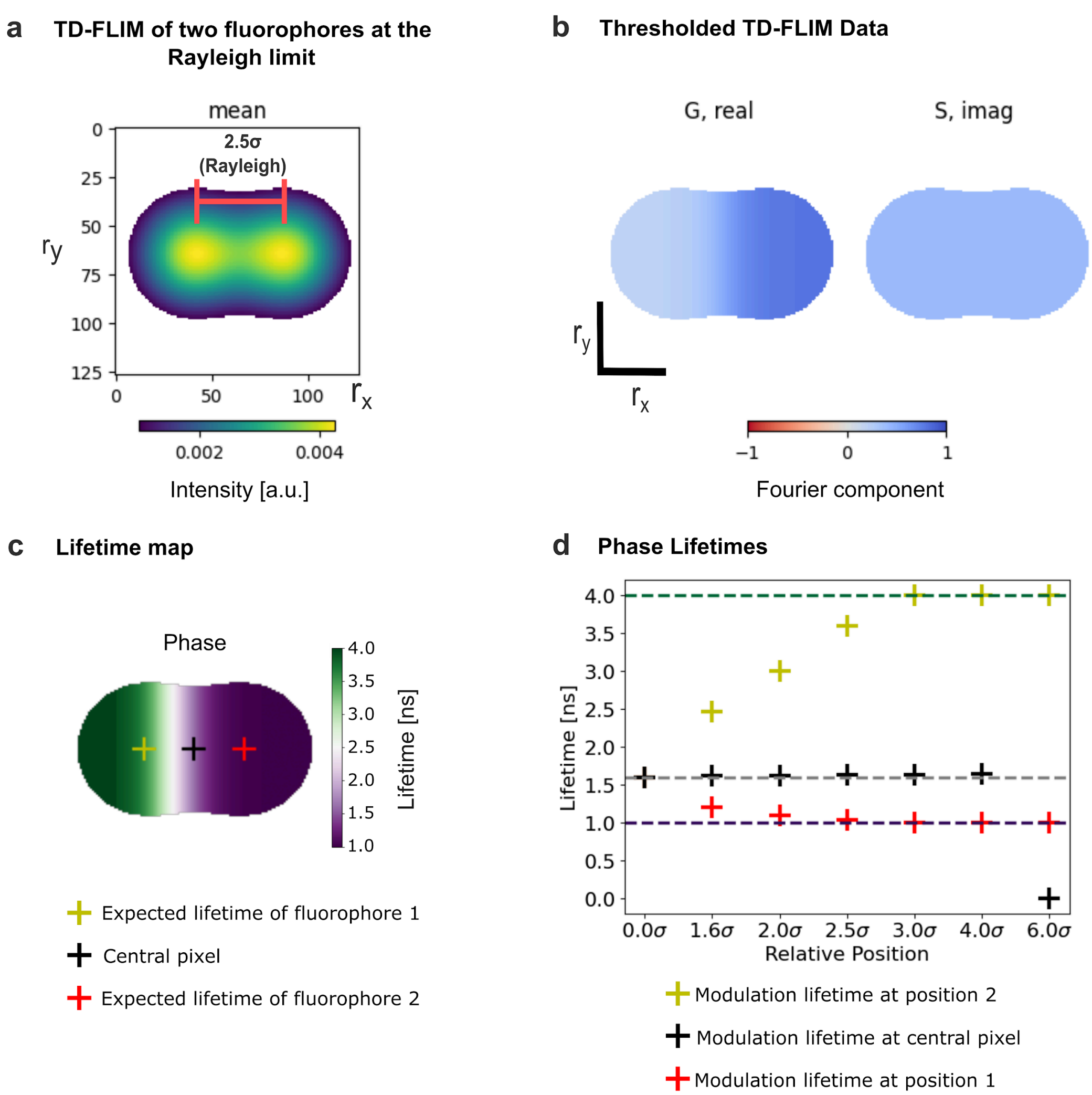

Fig. S2. Simulated TD-FLIM dataset for two independent, non-interacting fluorophores positioned at 2.5σ**.** The phase lifetime map is displayed alongside the corresponding spatial intensity reference. Panels (a–c) show simulated TD-FLIM images of two fluorophores positioned at 2.5σ. (a) Steady-state fluorescence intensity (mean from Fourier analysis) with intensity thresholding applied to suppress background. Axes represent pixel coordinates (r_x, r_y). (b) Phasor component maps: G (real) and S (imaginary). (c) Phase lifetime map computed from Fourier components. The transition zone in phase lifetime is shifted toward the long-lifetime fluorophore relative to the geometric midpoint of the two emitter positions. (d) Phase lifetimes sampled at the centers of each fluorophore position and at the central reference pixel, plotted as a function of inter-fluorophore separation (σ).

S3. FD-FLIM *in silico* simulation of Temporal Blending induced by PSF overlap.

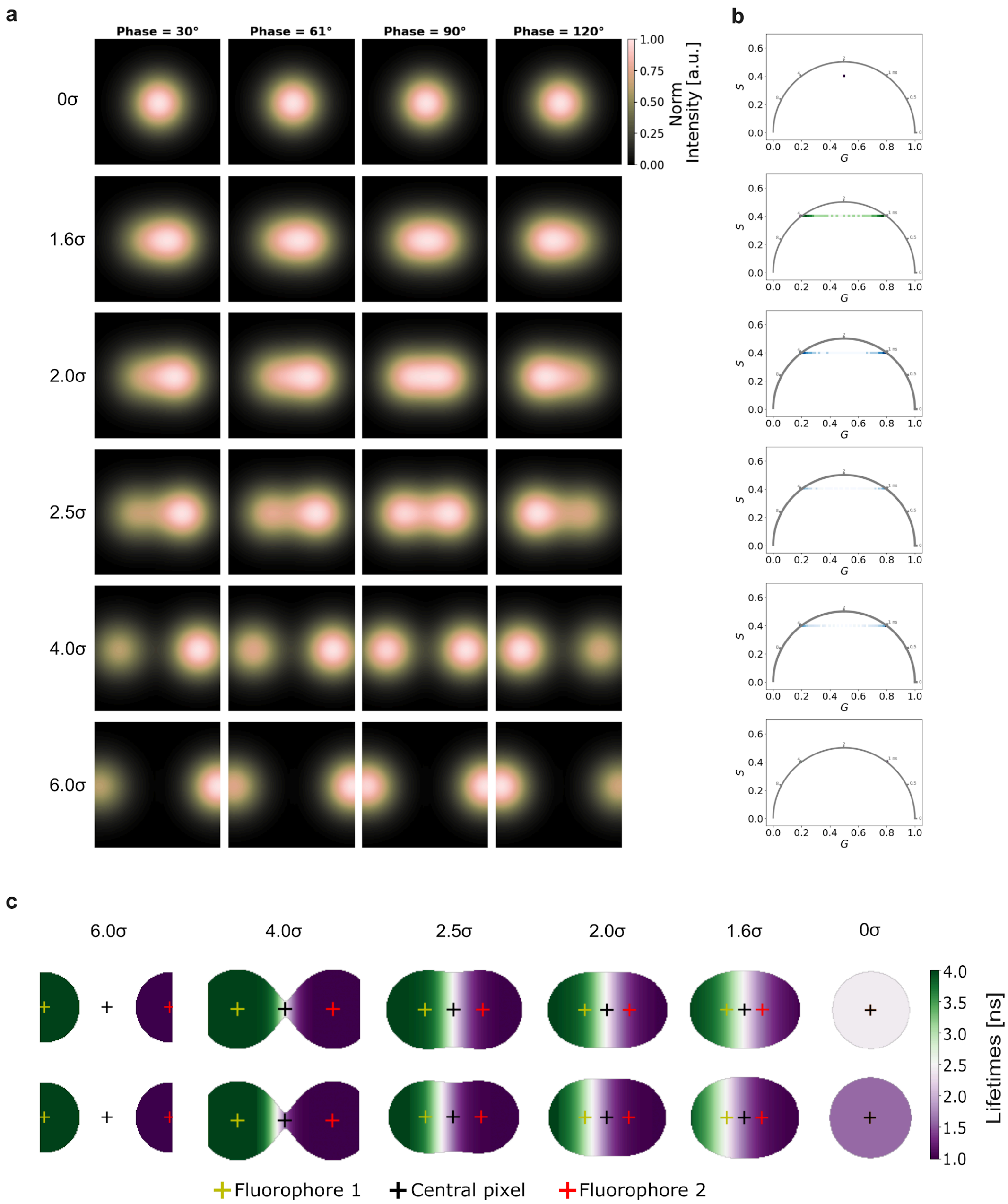

Fig. S3. Frequency-domain implementation of a two-fluorophore *in silico* model consisting of two independent, non-interacting and monoexponential emitters with lifetimes of 4ns (left) and 1ns (right).(a) Representative sinusoidally modulated gaussian-blurred intensity frames at four relative phase angles (30°, 60°, 90°, and 120°). Each row corresponds to a different inter-fluorophore separation expressed in units of the Gaussian PSF standard deviation σ, ranging from complete overlap (0σ) to well-separated emitters (6σ). As the separation decreases, PSF-induced spatial mixing becomes increasingly pronounced. (b) Corresponding phasor plots derived from the data in panel (a). With decreasing inter-fluorophore distance, phasor clusters progressively broaden and shift toward an amplitude-weighted mixed position inside the universal semicircle, reflecting apparent multi-exponential behavior arising solely from spatial averaging. This demonstrates that TB emerges in FD-FLIM through PSF-mediated photon mixing, even when the underlying fluorophores have strictly monoexponential decay. (c) Modulation- and Phase-lifetime maps associated with the simulated FD-FLIM data, illustrating how spatial overlap produces intermediate apparent lifetimes in regions between emitters. Together, these simulations confirm that TB manifests analogously in FD-FLIM as a geometric-optical effect rather than a change in intrinsic decay kinetics.

S4. Percentile-based MSSR masking progressively reduces Temporal Blending at the Sparrow limit.

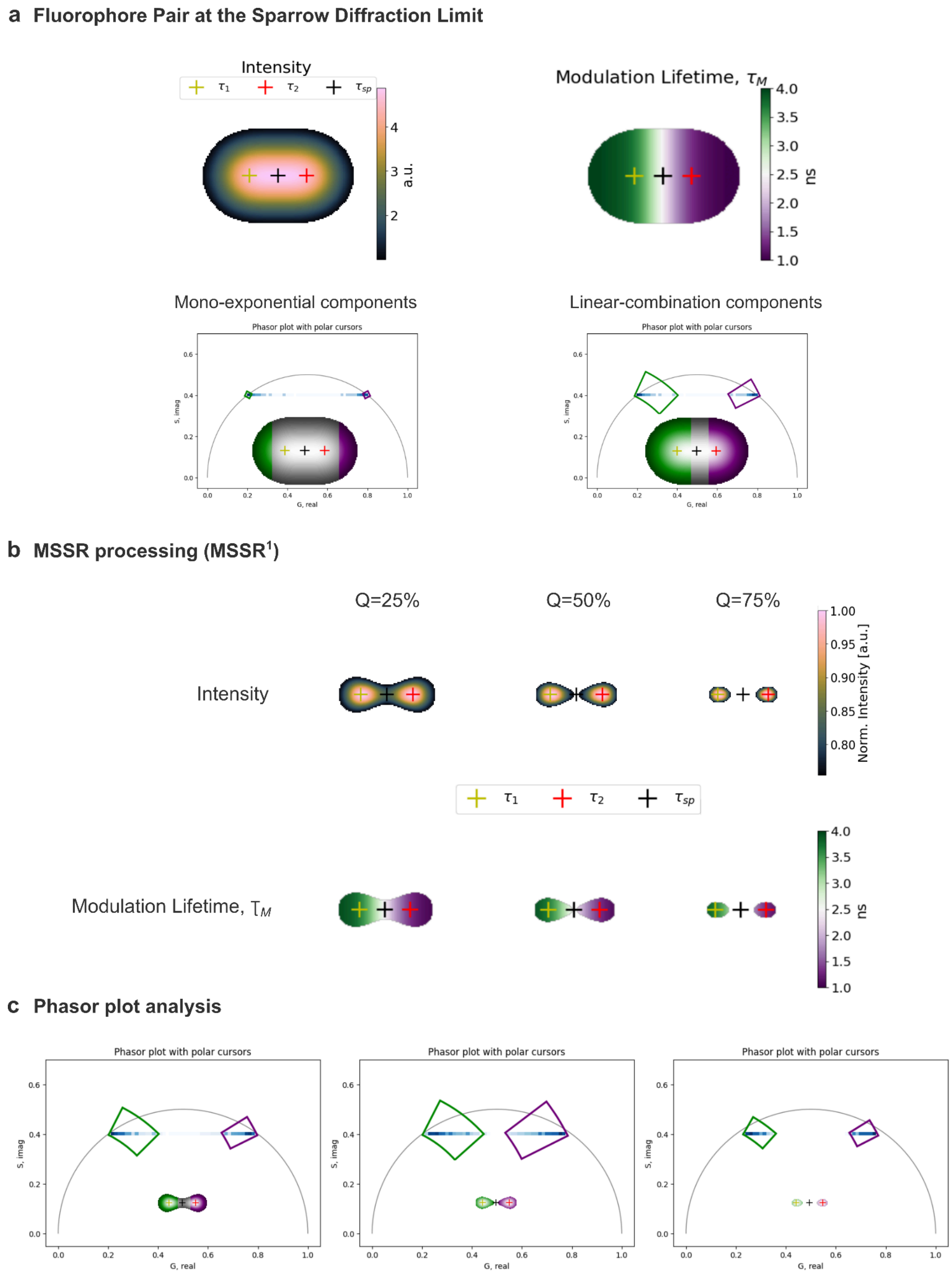

Fig. S4. Two independent, non-interacting fluorophores simulated at the Sparrow diffraction limit (2.0σ; same dataset as Fig. 8) are analyzed using MSSR-derived spatial probability masks. (a) Diffraction-limited intensity image, corresponding modulation lifetime map, and phasor plots illustrating monoexponential components (lying on the universal semicircle) and linearly combined components arising from PSF-induced overlap, the insets show selected pixel regions on the modulation lifetime maps that were segmented in the phasor space by the rectangle-polar cursors to illustrate with the reciprocity principle the extension difference of the monoexponential pixels and the linearly-combined pixels. (b) MSSR[1]-processed intensity images thresholded at the 25th, 50th, and 75th percentiles (top row), with the corresponding modulation lifetime maps shown below. Increasing the percentile threshold progressively restricts the analysis to spatial regions with a higher probability of originating from individual emitters, thereby reducing effective PSF overlap. (c) Phasor analysis of the MSSR-masked datasets reveals a monotonic reduction in phasor cluster spread and a depletion of intermediate apparent lifetimes as the threshold increases. In contrast, the monoexponential phasor positions remain stable. Together, these results demonstrate that percentile-based MSSR masking systematically mitigates Temporal Blending by spatially excluding mixed pixels without altering the intrinsic fluorescence decay signatures.

S5. Phasor Cloud and Fraction Analysis Metrics

To quantify the effect of MSSR on the phasor cloud, we computed several statistical descriptors from the 2D distributions of the real (G) and imaginary (S) components. Pixels below the applied intensity threshold were excluded (NaN values ignored).

Metric	Expected Values / Behavior	Interpretation
Centroid displacement (Δr)	≈ 0 (no displacement)	Preservation of intrinsic lifetimes. Large displacements (>0.05 in normalized phasor space) indicate distortion of decay kinetics information.
Ellipse area ratio (A_{after}/A_{before})	< 1	Ratio < 1: reduced phasor cloud spread.<br>Ratio ≈ 1: no effect.<br>Ratio > 1: increased uncertainty or artifact introduction.
Transition slope	< 0	More negative values indicate a steeper transition between lifetime populations.

Table S1. Quantitative metrics to measure changes in the phasor analysis due to MSSR-masking

Metric	Equation
Centroid	$(\bar{G}, \bar{S}) = (\frac{1}{N}\sum_{i=1}^{N} G_i, \frac{1}{N}\sum_{i=1}^{N} S_i)$
Centroid displacement	$\Delta r = \sqrt{(\bar{G}_{after} - \bar{G}_{before})^2 + (\bar{S}_{after} - \bar{S}_{before})^2}$
Ellipse area (95% confidence)	$A = ab\pi$,<br>$a = \sqrt{\lambda_1 \chi_\alpha^2}, b = \sqrt{\lambda_2 \chi_\alpha^2}; \lambda_1, \lambda_2 = eig(\Sigma); \lambda_1 \geq \lambda_2$<br>$\chi_\alpha^2 = \chi^2_{0.95,2} = 5.991$
Ellipse area ratio	$R_A = \frac{A_{after}}{A_{before}}$
Fraction contribution transition slope	$m = \frac{\Delta N}{\Delta f}$

Table S2. Interpretation of metrics

These metrics evaluate MSSR's performance in enhancing the precision of lifetime estimation in phasor space without introducing artifacts. Centroid stability demonstrates preservation of intrinsic lifetime signatures, reduction in ellipse area indicates suppressed temporal blending, and the slope in the fractional contribution measures the reduction of fractional contributions to lifetime populations. Together, these results provide quantitative evidence that MSSR improves both spatial and temporal fidelity in FLIM phasor analysis.

S6. Effect of MSSR masking on the phasor representation and fractional contribution of lifetime populations.

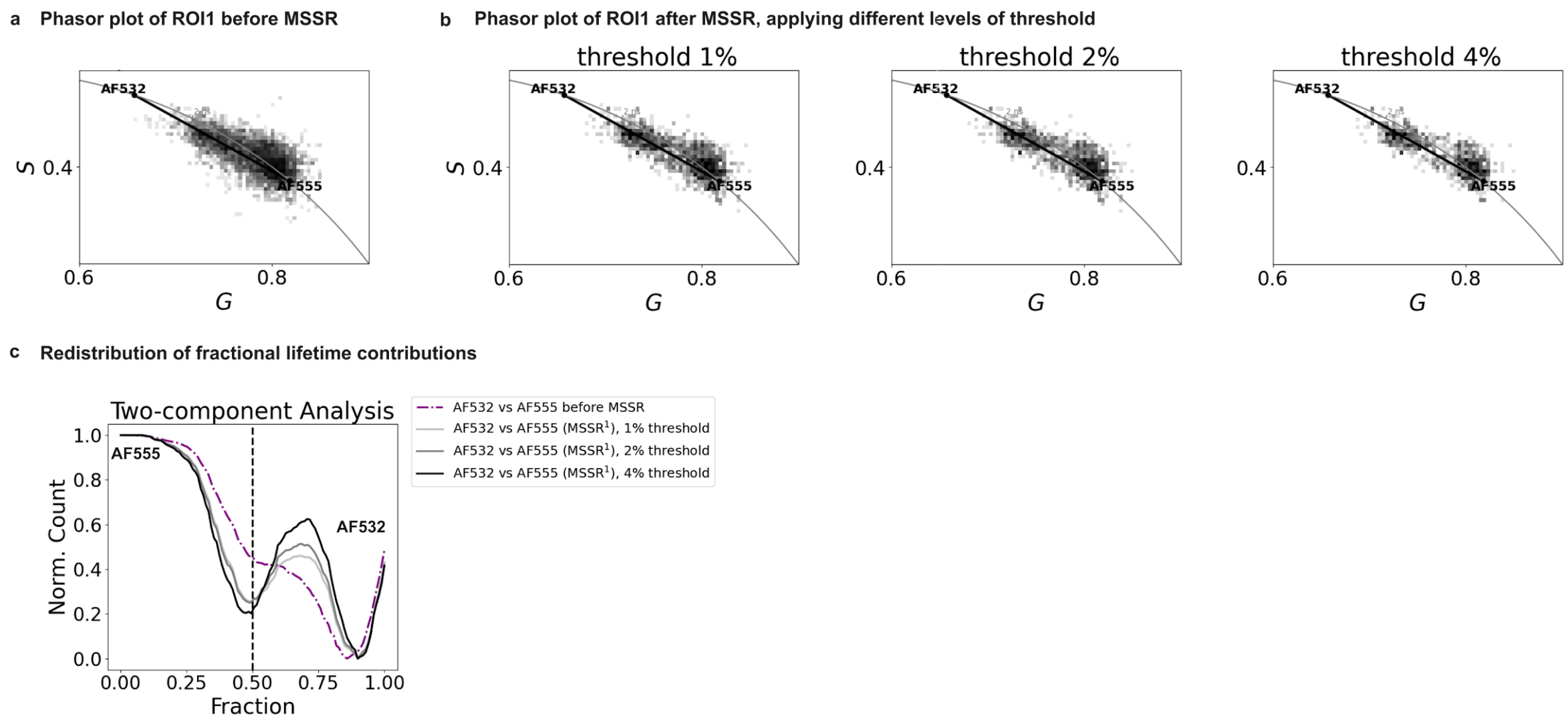

Fig. S5. (a) Phasor distribution of ROI1 before MSSR processing. Phasor points are distributed along the mixing trajectory between the reference lifetimes of AF532 and AF555, reflecting the contribution of mixed pixels within the diffraction-limited image. (b) Phasor distributions obtained after applying the MSSR-derived mask at three probability thresholds (1%, 2%, and 4%) before phasor analysis. (c) Two-component fractional contribution analysis for the phasor populations shown in (a) and (b). The vertical axis represents the normalized counts of pixels with different fractional contributions (horizontal axis). The dashed vertical line indicates an equal (50%) contribution of both fluorophores. The increase in the counts at ~0.75 fraction is expected by the enrichment of the AF555 population due to pixel retention by the MSSR masking.

Table S3. Results of slope estimation in the fractional contribution plots for the 2-color FLIM case.

Plot	Slope	Ratio (m_{After}/m_{before})
Before MSSR	-4.8×10^{-2}	
1% Threshold	-8.0×10^{-2}	1.66
2% Threshold	-8.5×10^{-2}	1.77
4% Threshold	-9.3×10^{-2}	1.94

S7. References

1. Digman, M. A.; Caiolfa, V. R.; Zamai, M.; Gratton, E. The Phasor Approach to Fluorescence Lifetime Imaging Analysis. *Biophys. J.* **2008**, *94* (2), L14–L16. https://doi.org/10.1529/biophysj.107.120154.

2. Ranjit, S.; Malacrida, L.; Jameson, D. M.; Gratton, E. Fit-Free Analysis of Fluorescence Lifetime Imaging Data Using the Phasor Approach. *Nat. Protoc.* **2018**, *13* (9), 1979–2004. https://doi.org/10.1038/s41596-018-0026-5.

3. Malacrida, L.; Suman Ranjit; Jameson, D. M.; Gratton, E. The Phasor Plot: A Universal Circle to Advance Fluorescence Lifetime Analysis and Interpretation. *Annu. Rev. Biophys.* **2021**, *50* (1), 575–593. https://doi.org/10.1146/annurev-biophys-062920-063631.

4. Torrado, B.; Malacrida, L.; Suman Ranjit. Linear Combination Properties of the Phasor Space in Fluorescence Imaging. *Sensors (Basel)* **2022**, *22* (3), 999–999. https://doi.org/10.3390/s22030999.

5. Malacrida, L. Phasor Plots and the Future of Spectral and Lifetime Imaging. *Nat. Methods* **2023**, *20* (7), 965–967. https://doi.org/10.1038/s41592-023-01906-y.

6. Yuan, X.; Varun Mannam; Zhang, Y.; Howard, S. Overcoming the Fundamental Limitation of Frequency-Domain Fluorescence Lifetime Imaging Microscopy Spatial Resolution. *Proc. SPIE 11650* **2021**. https://doi.org/10.1117/12.2577284.
